# ﻿Four new species of *Phytocoris* Fallén (Hemiptera, Miridae) from the Davis Mountains in Texas and further documentation of known species of Jeff Davis County

**DOI:** 10.3897/zookeys.1174.107083

**Published:** 2023-08-08

**Authors:** Katrina L. Menard, Michael D. Schwartz

**Affiliations:** 1 Department of Ecology & Evolutionary Biology, University of Connecticut, Unit 3043, 75 N. Eagleville Rd, Storrs, CT 06269-3043, USA University of Connecticut Storrs United States of America; 2 Canadian National Collection, AAFC, CEF, Ottawa ON K1A 0C6, Canada Canadian National Collection Ottawa Canada

**Keywords:** Biodiversity, Davis Mountains, Miridae, new species, USA

## Abstract

A recent survey of the entomofauna of the Davis Mountains in the state of Texas has revealed four new species in the genus *Phytocoris* Fallén (Miridae, Mirinae, Mirini): *Phytocorismcivor***sp. nov.** and *Phytocorisschmitzi***sp. nov.** found on *Quercusgrisea* Liebmann, and *Phytocorismarqua***sp. nov.** and *Phytocorisrileyi***sp. nov.** found attracted to lights. Descriptions, habitus, and genitalic images for the new species are included herein. Further, habitus and genitalic photographs of known *Phytocoris* species from the county are included to aid in identification.

## ﻿Introduction

The Davis Mountains in Jeff Davis County, Texas, USA, is a unique geological feature in west Texas that encompass several different ecosystems and are the second tallest mountains in the state behind the Guadalupe Mountains in Culberson County. The summit, Mount Livermore, reaches more than 8378 feet, allowing it to maintain precipitation levels and plant fauna characteristic of other “sky islands” that are surrounded by arid environments (Nature Conservancy 2008). At lower elevations there is diverse plant fauna that otherwise is only found in other “sky islands” in the west, such as in Arizona, New Mexico, Utah, and Colorado. Additionally, there are many endemic plants only found in that mountain range and records of plants found nowhere else in Texas (Keeling 2017).

An arthropod-based biodiversity survey of the Davis Mountains was undertaken at the largest land conservation area, the Nature Conservancy Davis Mountains Preserve, in 2020. Spearheaded by the entomologist Ashley Schmitz, the survey has a multi-year goal to document as much of the arthropod diversity as possible. The lead author was brought in to help identify the Heteroptera fauna being collected (Insecta, Hemiptera), and document possible new state records or new species on the endemic plants found nowhere else in the state. Collecting on grey oak (*Quercusgrisea* Liebm.) adjacent to the Nature Conservancy McIvor Center and further south on *Quercusgravesii* Sudworth, two series of *Phytocoris* Fallén were found that did not key out to any currently known species. Subsequent review of other *Phytocoris* specimens collected from Jeff Davis County held at the Texas A&M University Entomology Collection (TAMU) led to the discovery of two other unknown species collected at lights from private property on the other side of the Davis Mountains.

*Phytocoris* the most specious genus in the family Miridae with more than 700 described species (Schuh 2002–2013). The genus has a Holarctic distribution, and several works have focused on the Nearctic fauna north of Mexico. Early workers such as Uhler (1894), Reuter (1876, 1908, 1909, 1912), Van Duzee (1914, 1916, 1917, 1918, 1920, 1923), and Johnston (1930, 1935) described four, 36, 31, and two species, respectively. Knight (1920, 1923, 1925, 1926, 1927, 1928, 1934, 1941, 1961, 1968, 1974), however, was the most prolific with describing at least 116 species during a 60-year span. He also was instrumental in grouping the taxa into four tentative species groupings, primarily based on color patterns of the hemelytra, wing membrane and antennae, length of antennal segment I, setal vestiture, and shape of the endosomal sclerites in the males (Knight 1923, 1941). Other miridologists subsequently described additional species of *Phytocoris* from North America such as Bliven (1956, 1959, 1966), Kelton (1979), Henry (1974, 1979, 1985, 2009), as well as Schwartz and Chérot (2005), some of whom utilized Knight’s framework to contextualize their species relationship to other taxa. Stonedahl also described many species (Stonedahl 1984, 1988, 1995), and expanded on Knight’s hypothesis with his monograph of the western species (Stonedahl 1988), which included investigating morphological diversity of the species across the genus, the phylogeography of the western North American taxa, and expanding the species groups to 20.

Focusing on west-Texas *Phytocoris* species, Stonedahl (1988) found a total of 53 species endemic to southeastern New Mexico and west Texas, later including a further 11 species described from additional TAMU material from Texas (Stonedahl 1995). The four new species herein belong to the *junceus*, *hopi*, and *rostratus* groups proposed by Stonedahl (1988), the same groups Stonedahl (1995) found closely aligned to his other Texan species. Because habitus illustrations and photographs are not available for most taxa from this region of Texas and illustrations of the male genitalia are only partial, we have also included diagnoses of known taxa from this county including habitus and endosomal photographs.

## ﻿Materials and methods

Approximately 200 specimens were examined from fieldwork conducted by KLM in Texas as well as from material from various museums, including a synoptic set of *Phytocoris* species determined by Gary Stonedahl as part of his 1988 and 1995 works given to MDS at the Canadian National Collection. The undetermined *Phytocoris* sp. specimen collections from Texas A&M University from west Texas (Jeff Davis, Presidio, Brewster, Pecos, Culberson, and Reeves counties) were also surveyed for additional undescribed material in the series.

The following abbreviations are used for institutions cited in this paper:

**AMNH**American Museum of Natural History, New York, New York, United States; Ruth Salas;

**CNC**Canadian National Collection, Ottawa, Canada; Michael Schwartz;

**TAMU**Texas A & M Entomology Collection, College Station, Texas, United States; Karen Wright;

**UCONN**University of Connecticut, Storrs, Connecticut, United States; Katrina Menard;

USNM United States National Museum, Washington, District of Columbia, United States; Thomas Henry.

Terminology for male genitalia follows Stonedahl (1988) and Schwartz (2011). Genitalia were extracted by soaking the abdomen in diluted potassium hydroxide (KOH) or an 85% lactic acid solution for one to a few days at room temperature, and then washing with distilled water before being dissected in glycerol.

Dorsal habitus images and genitalic images were taken with a JVC C-mount K Y-F75u digital camera attached to a Leica MZ16 dissecting microscope or an Olympus BX51 compound microscope using Auto-montage Pro (v. 5.02) image stacking software installed on a Dell Precision T1500 computer operating with Windows XP. Stacked images were combined using the compound weighted algorithm optimized for precision to obtain full depth of view habitus images.

Measurements were taken using an eyepiece micrometer (10 mm/100) on an Olympus SZX2 stereomicroscope and are shown in Table 1. Measurements include the body length from the apex of the clypeus to the apex of the membrane, the width of the head across eyes, the interocular width (vertex), the length of the labium, the lengths of the antennal segments I–IV, the median length of the pronotum (anterior margin to posterior margin), the posterior width of the pronotum (widest point across the posterior margin), and the width across the widest point of the hemelytra. All measurements are in millimeters.

**Table 1.** Measurements of described new species of *Phytocoris* Fallén.

Measurements (in mm).											
Species		Length						Width			
		Clyp-Memb.	Pronotum	Ant1	Ant2	Ant3	Ant4	Head	Pronotum	Hemelytra	InterOcDi
***P.marqua* sp. nov.**											
M (*n* = 3)	**Mean**	**3.96**	**0.44**	**0.64**	**1.16**	**0.27**	**0**	**0.44**	**0.77**	**1.33**	**0.2**
	SD	0.26	0	0.04	0.04	0.46	0	0	0.02	0.07	0.01
	Min	3.75	0.44	0.6	1.12	0	0	0.44	0.76	1.25	0.2
	Max	4.25	0.44	0.68	1.2	0.8	0	0.44	0.8	1.38	0.2
***P.mcivor* sp. nov.**											
M (*n* = 2)	M1	4.875	0.48	0.68	1.4	0.68	0.56	0.6	0.88	1.625	0.16
	M2	4.875	0.48	0.64	1.32	0.64	0.56	0.56	0.8	1.5	0.14
F (*n* = 2)	F1	4.125	0.4	0.52	1.24	0.56	0.44	0.52	0.72	1.4375	0.18
	F2	4	0.4	0.56	1.2	0.6	0.4	0.5	0.76	1.375	0.18
***P.rileyi* sp. nov.**											
M (*n* = 5)	**Mean**	**5.75**	**0.56**	**1.06**	**1.86**	**1.06**	**0.28**	**0.45**	**0.84**	**1.31**	**0.24**
	SD	0.19	0.01	0.02	0.03	0.02	0.38	0.02	0.02	0.04	0.01
	Min	5.5	0.54	1.04	1.84	1.04	0	0.44	0.8	1.25	0.22
	Max	6	0.58	1.08	1.9	1.08	0.72	0.48	0.86	1.375	0.26
F (n = *1*)		4.38	0.4	0.84	1.72	1.08	0.6	0.46	0.54	1.12	0.24
***P.schmitzi* sp. nov.**											
	Male	4.5	0.42	0.82	1.52	0.88	0.6	0.46	0.68	1.25	0.22
	Female	5	0.44	0.88	1.68	0.92	0.6	0.48	0.76	1.44	0.24

For species distribution records in Jeff Davis County, direct observations were made of specimens from the TAMU or CNC collections and verified by KLM and MDS, or directly collected by KLM or Ashley Schmitz in the Davis Mountains Nature Conservancy. For in-depth diagnoses, illustrations of the male genital capsule and parameres, and additional information about the previously described taxa, please refer to Stonedahl (1988, 1995).

## ﻿Results

### ﻿ Phytocoris marqua sp. nov.

Taxon classificationAnimaliaHemipteraMiridae

36F04496-3EB4-5A66-B9B7-BD027470A855

https://zoobank.org/A38C21B3-6EA0-424A-B1E9-24E3D3FB035E

Fig. 1

#### Type material.

***Holotype*** male, pinned. Original label: “United States: Texas: Jeff Davis Co., Davis Mts. Resort, 5,800 ft. (Marqua residence), UV, 30.62842°N, 104.08360°W, VII-4-5-09, E & M. L. Riley, TAMU-ENTO X0615825” (TAMU). ***Paratypes***: Same collecting event as holotype; X0615711 (USNM) 1♂; X0615790 (CNC) 1♂; X0615785 (UCONN) 1♂.

#### Diagnosis.

This species shares the diagnostic characteristics of the *rostratus* group, particularly the brown to dark brown coloration, the subquadrate head with a broad vertex, a dorsum with narrow, dark, scale-like setae mixed with other types of vestiture, and male genitalia with greatly reduced primary membranous sac of endosoma with two sclerotized processes and patches of spinules on the basal lobes. It is unique, however, in the combination of characteristics of the male genitalia including: the tapered and narrow shape of the right sclerite of the male endosoma, and the lack of spines on the left paramere.

#### Description.

**Figure 1.**
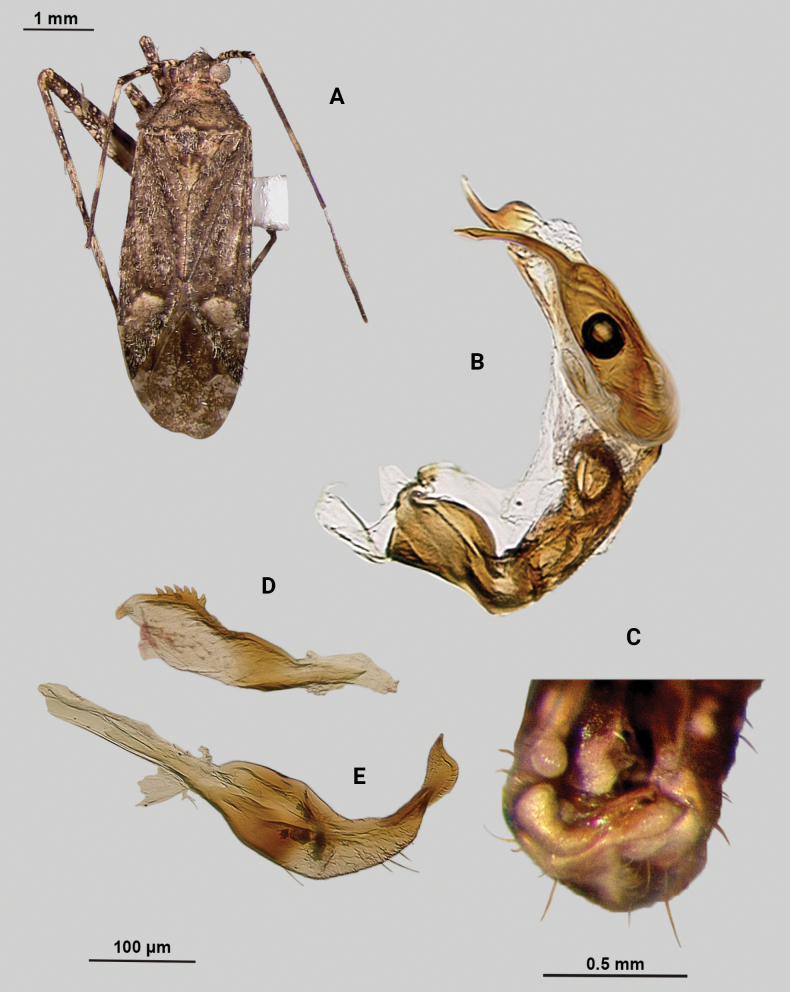
*Phytocorismarqua* sp. nov. **A** male dorsal habitus **B** male endosoma **C** male genital capsule in dorsal view **D** right paramere in lateral view **E** left paramere in lateral view.

**Male** (*n* = 3): Macropterous, medium sized, lateral margins weakly convex. Total length clypeus to apex membrane from 3.75–4.25 mm, widest point across hemelytra 1.25–1.38 mm; general coloration dark brown (Fig. 1A).

**Coloration. *Head***: predominantly dark brown in dorsal view, vertex with two parallel pale spots adjacent to eye margins at medians, frons with macula at approximate midline and anterior to lateral spots by eyes, area between antennal fossae and anterior margin of eyes also with narrow pale band, clypeus with narrow pale horizontal band on burgundy-brown background that transverses clypeus and genae medially in frontal view, maxillary plate with burgundy-brown stripe extending medially across into primarily whitish colored gena in lateral view, buccula burgundy-brown extending with darker coloration patterning along ventral margin of head including xyphus giving appearance of two horizontal dark stripes, labrum golden brown; labium basally burgundy on first segment, distally pale yellow-white on remaining segments; antennal segment I primarily dark burgundy-brown with pale maculae along anterior 2/3 of length and remaining length dark burgundy-brown, segment II with relatively narrow white band basally and wider band medially with remaining coloration dark brown, segment III with narrow pale band basally and remainder coloration dark brown, segment IV completely dark brown; eyes brownish burgundy. ***Thorax***: dorsal 1/2 of propleura dark brown and whitish yellow on ventral 1/2, mesopleura dark brown excluding white margins along mesocoxa, metapleura dark brown with ostiolar peritreme and metathoracic spiracle whitish yellow, anterior of pronotum and collar whitish grey with two dark brown stripes extending anteriorly along lateral angles of calli onto collar, posterior pronotum primarily pale brown with irregular dark brown line weakly connected to darker lateral stripes on anterior portion contrasting with whitish coloration of pronotal posterior margin, mesoscutum dark brown with lateral 1/4 areas whitish yellow, scutellum primarily whitish yellow with dark brown inverted triangle-shape corresponding to brown area of mesoscutum along dorsal 1/2, distal 1/2 variously mottled with dark brown spots; all coxae pale yellowish white with metacoxae possessing dark brown spot on anterior-basal surface, all femora pale whitish yellow basally with increasing coverage of burgundy-brown maculae distally particularly on metafemora, tibiae primarily whitish yellow with burgundy-brown bands of maculae at basal and anterior margins as well as two defined bands medially to form four defined annuli, all tarsal segments dark brown. ***Hemelytra***: predominantly greyish brown with darker blackish brown diffuse stripes along claval vein, cubitus, and costal vein, with dark and pale checkerboard pattern along costal margin and interior margin of cuneus adjacent to membrane, corium adjacent to medial 2/3 of cuneus with large whitish yellow patch extending distally into antero-lateral 1/3 of cuneus, with remaining cuneus dark brown with increasing number of blackish maculae distally towards membrane, membrane primarily comprised of brown maculae excluding two white patches along lateral margins adjacent cuneus apex, veins brown to dark brown. ***Abdomen***: burgundy-brown, apex of lateral tubercle and distal margins of genital segment transitioning to greyish white coloration.

**Surface and vestiture**. Dorsal surface of head, pronotum, mesoscutum, scutellum and hemelytra with long black erect setae mixed with silvery-sericeous setae, hemelytral surface texture otherwise matte.

**Structure**. ***Head***: clypeus not visible in dorsal view, vertex relatively rounded and slightly declining posteriorly and dorsal surface of eyes not surpassing vertex in lateral view, eyes relatively small with total height slightly < 1/2 total height head in lateral view, interocular distance slightly < 1/2 total width head, antennal segment I length > 1/2 length II antennal segment and width of head, apex of labium extending to hind coxae. ***Thorax***: collar well-developed, pronotum trapezoidal in shape with developed calli and posterior margin weakly concave, mesoscutum and scutellum relatively flat with declining margins towards clavus, legs relatively narrow with hind legs elongate, pretarsi with convergent parempodia and developed pulvillus. ***Hemelytra***: lateral margins weakly convex on distal 1/2, cuneus with angled declivity. ***Abdomen***: < 1/2 of total body length, apex not reaching apex of wing membrane, genital capsule relatively large, nearly 1/2 total length of abdomen. ***Genitalia***: Genital capsule with a well-developed tubercle on left margin weakly pointed at apex (Fig. 1C). Endosoma with elongate, relatively narrow membrane and two medially located well-developed sclerites; right sclerite basally rounded, distally narrowed with arrow-like shaped apex, attached to basal process below the secondary gonopore; left sclerite relatively wide terminating abruptly into narrow spine-like process pre-apically (Fig. 1B). Left paramere C-shaped with duck-head shaped apex (Fig. 1E). Right paramere leaf-shaped with hook-like apex and six serrated “teeth” on dorsal margin (Fig. 1D).

**Female.** Unknown

**Measurements.** Table 1.

#### Hosts.

Unknown; collected at a light sheet.

#### Distribution.

Davis Mountains, Jeff Davis County, Texas.

#### Etymology.

Named for the late entomologist David Marqua, whose property in the Davis Mountains yielded this unique taxon during light sampling. Noun in apposition.

#### Remarks.

This species is most similar to *Phytocoriskerrvillenis* Stonedahl based on the shape of the right paramere (particularly the serrations on the dorsal margin), the well-developed tubercle, the rounded base of the right sclerite of the endosoma, and the reduced primary membrane. However, it can easily be separated by the different dorsal patterning, including the completely pale grey scutellum, completely dark brown posterior margin of the pronotum in *P.kerrvillenis* versus the dark and grey patterning in *P.marqua*. Further, the differences in the spines on the left paramere that are present on *P.kerrvillenis* but absent in *P.marqua*, and the tapered and narrow portion of the right sclerite in *P.marqua* that is relatively squat and broad in *P.kerrvillenis* separate the two.

Stonedahl (1988) initially proposed the *rostratus* species group for taxa that are united by the following characters: brown to dark brown coloration; head usually subquadrate to slightly elongate and strongly produced frons and tylus base, and broad vertex; length of the eye of male subequal to width of vertex except as noted in the species key; dorsum with narrow, dark, scalelike setae mixed with other types of vestiture; and male genitalia with greatly reduced primary membranous sac of endosoma with two sclerotized processes and patches of spinules on the basal lobes. *Phytocorismarqua* fits these characteristics, but Stonedahl (1995) further refined his concepts of species relationships when conceptualizing his newly described taxa from Texas, *P.kerrvillenis*, and *P.davisi*. He grouped these taxa with previously described eastern taxa *P.albifacies*, *P.corticevivens*, *P.fumatus*, *P.sulcatus*, and *P.tuberculatus* of Knight’s (1923, 1941) “group 1” based on the following: head mostly pale ventrad of eyes; head with broad, dark band anteriorly stretching uninterrupted between antennal fossae; pronotal collar with series of stout, dark setae dorsally; pronotum with prominent, medially confluent calli, genital capsule with strongly developed tubercle above base of left paramere; endosoma of male with single, elongate primary membranous sac often with spines distally; sclerotized process of endosoma elongate, usually twisted and/or with reflexed margins. *Phytocorismarqua* fits all these characteristics as well, suggesting it is closely related to this possible monophyletic group though a cladistic analysis should be performed to support this hypothesis.

### ﻿ Phytocoris mcivor sp. nov.

Taxon classificationAnimaliaHemipteraMiridae

73EA72CF-B03F-5876-B41F-02A8C05431C3

https://zoobank.org/58A60EAC-2876-4213-A289-5C3C71B1FF50

Fig. 2

#### Type material.

***Holotype*** male, pinned. Original label: “United States: Texas: Jeff Davis Co.: Davis Mountains Nature Preserve, McIvor Conservation Center, 30.694554 -104.121378, 5902 ft, 17.v.2022, K Menard, UV light” (TAMU). ***Paratypes***: United States: Texas: Jeff Davis Co., Davis Mountains Resort, 5800 ft, (Marqua residence), UV, 30.62842°N, 104.08360°W, 14.x.2011, EG Riley, X0893871 (TAMU), 1 ♂; same collecting event as holotype (USNM), 1♂; Davis Mountains Nature Preserve, McIvor Conservation Center, 30.694554, -104.121378, 5902 ft, 17.v.2022, K Menard, found on *Quercusgrisea* (Fagaceae) (UCONN), 1♂ 1♀.

#### Diagnosis.

Recognized by the following characteristics of the *junceus* species group, but unique in lacking a blunt tubercle on the male genital capsule: the large, elongate body form; short, elliptical head with large eyes, the slightly shiny dorsal surface, vestiture and scutellum without scale-like setae, the elongate right paramere, and the large and coarsely serrate sclerotized process of the endosoma.

#### Description.

**Figure 2.**
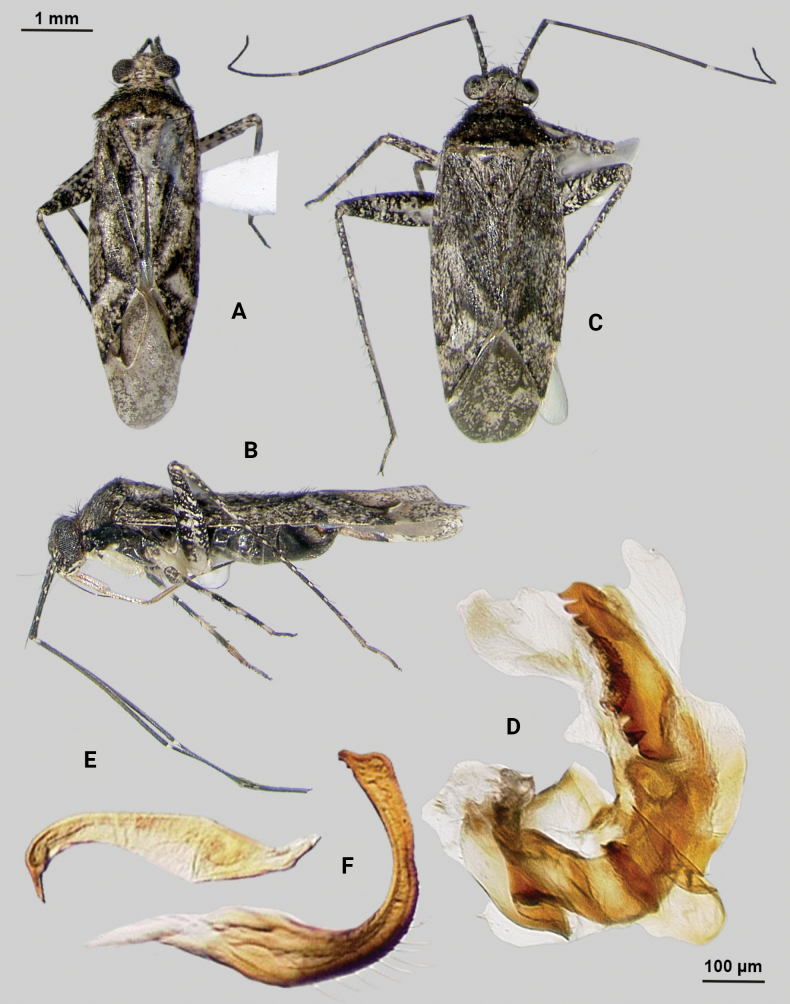
*Phytocorismcivor* sp. nov. **A** male dorsal habitus **B** male lateral view **C** female dorsal habitus **D** endosoma **E** right paramere in lateral view **F** left paramere in lateral view.

**Male** (*n* = 3): Macropterous, medium sized, lateral margins parallel-sided. Total length apex to apex membrane 4.63–4.88 mm, widest point across hemelytra 1.5–1.63 mm; general coloration dark brown (Fig. 2A).

**Coloration. *Head***: yellowish white with dark brown to black mottling across vertex and frons increasing in coverage ventrally, apical portion dark brown and ventral margins of clypeus, maxillary plate, and buccula white, labrum golden-brown; first segment of labium pale with thin red stripe along length, second and third segments yellowish, apical segment dark brown; antennal segment I primarily dark brown to black with relatively widely distributed pale maculae along length, segments II and III dark brown with narrow band of white at basal joint with previous segments, segment IV completely dark brown; eyes brownish burgundy. ***Thorax***: dorsum of propleura dark brown on apical three-fourths with whitish coloration on ventral 1/4 adjacent to xyphus and part of procoxae, mesopleura dark brown excluding white margins of spiracle, metapleura dark brown with ventral ½ of ostiolar peritreme and all of spiracle whitish yellow (Fig. 2B), anterior of pronotum and collar whitish yellow with burgundy-brown maculation increasing in coverage distally towards posterior portion, posterior portion of pronotum primarily dark brown with medial horse-shoe-shaped area of paler coloration whose distal apices on posterior margin of pronotum transition to white coloration and interior margin of shape possesses irregular dark brown line adjacent to distal white line to form weak spine-like pattern in dorsal view, mesoscutellum dark brown with two lateral whitish yellow spots, scutellum primarily whitish yellow with two thin dark brown vertical lines adjacent to median that separate posteriorly to form inverted Y-shape pattern; all coxae pale yellowish white with dark brown spot on anterior-basal surfaces, all femora pale whitish yellow basally with increasing dense coverage of black maculae distally, pro-tibiae with basal margin pale yellowish white followed by three bands of dark annuli terminating in dark band at apex of tarsomeres, meso-tibiae matching in pattern to pro-tibiae except terminating in pale band at apex, meta-tibiae completely dark brown with partial white band on ventral surface medially, all tarsal segments dark brown. ***Hemelytra***: predominantly pale grey with dark grey and white patches and black mottling, clavus primarily dark grey on anterior 1/2 transitioning to large white patch mainly distributed along claval suture, corium grey with diffuse brown patches more defined in shape distally, costal margin with dark and pale checkerboard pattern, area of corium adjacent to medial 2/3 of cuneus with large whitish yellow patch extending distally into basal margin of cuneus, with remaining surface coloration of cuneus dark brown with white maculae, membrane with dense brown maculae and single white patches along lateral margins adjacent cuneus apex, veins brown to dark brown. ***Abdomen***: dark brown, margins of gonopore paler brown.

**Surface and vestiture.** dorsal surface of head, pronotum, mesoscutum, scutellum and hemelytra with long black erect setae mixed with silvery-sericeous setae and some dark setae in defined patches on apex of clavus and interior margin of cuneus, hemelytral surface texture relatively smooth and shiny. **Structure: *Head***: tylus and clypeus not visible in dorsal view, vertex relatively rounded and slightly declining posteriorly and dorsal surface of eyes surpassing vertex in lateral view, eyes relatively large with total height > 1/2 total height head in lateral view, interocular distance less than width of single eye, antennal segment I length greater than width of head, apex of labium extending to genital capsule. ***Thorax***: collar relatively thin but well developed, pronotum trapezoidal in shape with developed calli and posterior margin weakly concave, mesoscutum and scutellum relatively flat with declining margins towards clavus, legs relatively narrow with hind legs elongate, pretarsus with convergent parempodia and developed pulvillus. Hemelytra: lateral margins parallel sided, cuneus with shallow declivity. ***Abdomen***: ~ < 1/2 of total body length, apex not reaching apex of wing membrane, genital capsule relatively small being 1/3 total length of abdomen. ***Genitalia***: genital capsule without a tubercule on left margin of gonopore. Endosoma with primary membranous sac developed into three well developed lobes, two of whose apices extend past adjacent sclerotized process anterior margin; right sclerotized process overall shape cone-like, with serrated margin on interior lateral edge comprised of nine “teeth” curved medially, bean-shaped sclerotized patch adjacent to median with spine-like processes (Fig. 2D). Left paramere C-shaped with post-medial indentation on dorsal surface, broadening distally until abruptly terminating into ventrally oriented, hook-like apex (Fig. 2F). Right paramere elongate and narrow with angulate anterior curvature at apex also terminating into defined point (Fig. 2E).

**Females** (*n* = 2). Same as males in coloration, surface, vestiture and structure aside from being slightly smaller and having relatively wider interocular distance relative to width of head (Fig. 2C).

**Measurements.** Table 1.

#### Hosts.

*Quercusgrisea* Liebmann (Fagaceae).

#### Distribution.

Davis Mountains, Jeff Davis County and Brewster County, Texas.

#### Etymology.

Named for the McIvor Conservation Center of the Nature Conservancy’s Davis Mountains Preserve, where this species was discovered on an adjacent oak tree and light sheet. Noun in apposition.

#### Remarks.

*Phytocorismcivor* sp. nov. appears to be most closely related to *Phytocorisquercinus* Stonedahl and *Phytocorisintermontanus* Stonedahl of Stonedahl’s (1988)*junceus* species group, and in fact keys to the couplet separating the two. The apices of the tibiae are pale in coloration which should key it out to *P.quercinus*, but the shaft of the left paramere is narrow in *P.quercinus* whereas and has a “dip” medially in *P.mcivor*. Further, the right paramere in *P.quercinus* does not have the hook-like apex of *P.quercinus*. Lastly, *P.mcivor* only has eight serrations on the sclerotized process of the endosoma versus the 13 or more of *P.quercinus*. The other species, *P.intermontanus*, has dark apices of the tarsi and differs by the shape of the serrations of the sclerotized process, being larger, more elongate serrations particularly on the lower half of the structure despite the similar number as *P.mcivor*. The apex of the right paramere in *P.mcivor* is also more similar in shape to *P.intermontanus*, with the hook-like apex, but the curvature is more angulate. *Phytocorisdecurvatus* Knight also looks superficially similar to *P.mcivor* but can be separated by the cone-like shape of the sclerotized process and relatively wide right paramere. All four species also have host plants in the genus *Quercus* L. (Fagaceae), which differs from the primarily coniferous hosts of other members of the *junceus* species group (Stonedahl 1988).

As Stonedahl (1988) summarized, the *junceus* species group was established by Knight (1974) for a more widely distributed group of taxa beyond the Western United States and including taxa from southern Canada and the northern United States. These taxa are unified by the large, elongate body form; short, elliptical head with large eyes, the slightly shiny dorsal surface, vestiture and scutellum without scale-like setae, and the structure of the male genitalia, particularly the elongate right paramere, large and coarsely serrate sclerotized process of the endosoma, and the genital capsule usually with a large, blunt tubercle above the paramere bases. *Phytocorismcivor* possesses all these characteristics except the blunt tubercle, which is absent.

### ﻿ Phytocoris rileyi sp. nov.

Taxon classificationAnimaliaHemipteraMiridae

531E200A-DFA3-5415-BC1F-189283F8829C

https://zoobank.org/18F73A60-44FF-4F97-AD6B-F4D6217472F3

Fig. 3

#### Type material.

***Holotype*** male, pinned. Original label: “United States: Texas: Jeff Davis Co., Davis Mts. Resort, 5,800 ft, (Marqua residence), UV, 30.62842°N 104.0360°W, X-14-2011 E. G. Riley, X0891959” (TAMU). ***Paratypes***: United States of America: Texas: Jeff Davis Co. McDonald Observatory, 2135 m, 10.ix.1988, RS Anderson, (AMNH_PBI 00419795—AMNH_PBI 00419796) (CNC) 2♂; same collecting event as holotype, X0893720 (UCONN), X0894589, X0894161 (TAMU) 3♂. Brewster Co. BBNP, Pine Canyon Camp Area no. 4: 4,700 ft, 29°15'59"N, 103°14'04"W, 1.x.2005, Raber & Riley, 1♂ (TAMU); New Mexico: Catron Co.: Rt. 12 at Sand Flat Road (B032), 5 km NE Aragon, 2125 m, 17.ix.2015, 33°54.887'N, 108°30.320'W, AG Wheeler & TJ Henry, ex. *Baileya* sp.? (USNM), 1♂; Sandoval Co.: Rt. 4, 6.5 km N of Jemez Springs, 2050 m, 14.ix.2015, at Jaromillo Creek, 2600 m, 39°53.4637'N, 106°29.771'W, AG Wheeler & TJ Henry, ex. *Juniperusmonosperma* (USNM), 1♀.

#### Diagnosis.

Belonging to the *hopi* group and distinguished by the combination of the relatively large size, the overall pale-beige coloration of the hemelytra with dark brown stripes on lateral margins of the head and pronotum, the white medial line extending along the apex of the scutellum to the median of the tylus of head, the yellowish coloration of the claval vein, and the elongate shape of the right paramere.

#### Description.

**Figure 3.**
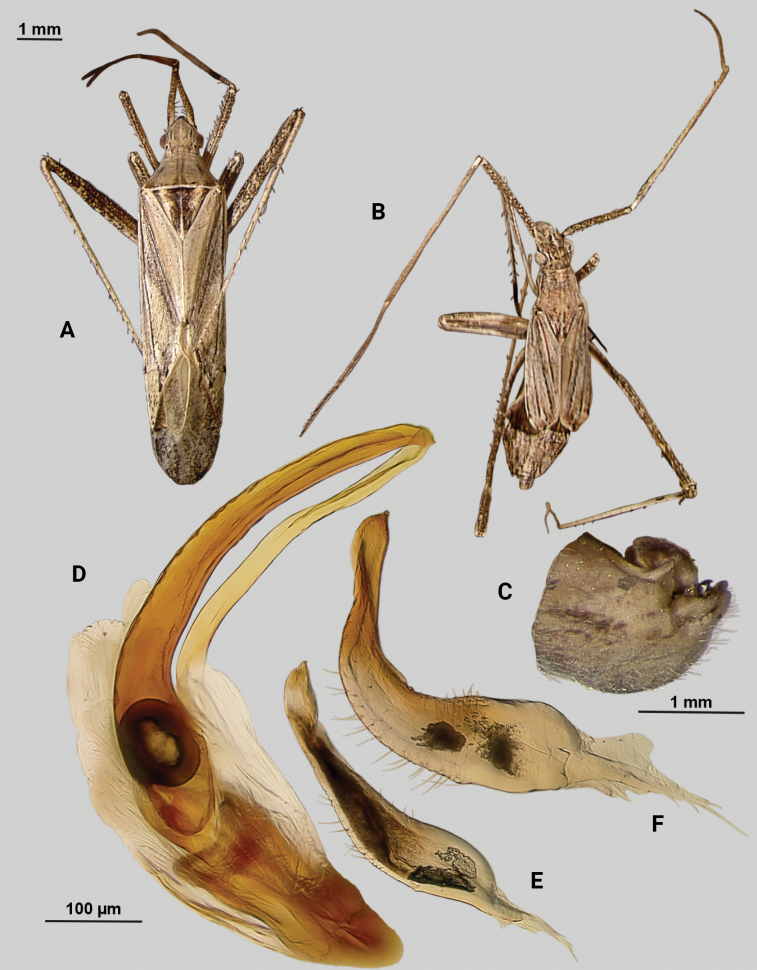
*Phytocorisrileyi* sp. nov. **A** male dorsal habitus **B** female dorsal habitus **C** male genital capsule in lateral view **D** endosoma **E** right paramere in lateral view **F** left paramere in lateral view.

**Male** (*n* = 5): Macropterous, relatively large, lateral margins weakly convex. Total length apex to apex membrane 5.5–6 mm, widest point across hemelytra 1.25–1.375 mm; general coloration beige and brown (Fig. 3A).

**Coloration. *Head***: primarily whitish beige with white midline stripe along length of head from base of vertex to median of tylus, dark narrow stripes along lateral margins of vertex as well as under eyes to antennal fossae, and horizontal dark stripe broken by vertical white stripe on top of vertex, frons with transverse dark markings associated with musculature attachment, maxillary plate whitish with dark ventral margin, buccula white, labrum white; first segment of labium pale, apical segments transitioning to dark brown; antennal segment I dark with dense white mottling and white stripe on latero-ventral surface extending three-fourths of basal length, segments II and III pale brown to beige with narrow band of white at basal joint with previous segments, segment IV completely dark brown; eyes pale pinkish brown. ***Thorax***: propleura whitish beige with three narrow horizontal dark brown stripes corresponding with stripes on lateral side of head, anterior-most stripe corresponding to lateral margin of pronotum, mesopleura whitish beige with dark brown horizontal stripe roughly corresponding to medial stripe on propleura and additional dark stripe ventrally, metapleura mostly dark brown with ventral 1/2 of ostiolar peritreme, spiracle margins, and coxal margins white, pronotal collar with two sets of narrow vertical brown lines with one set corresponding to dorsal brown stripe of propleuron and other dark stripes on vertex, collar and pronotum primarily whitish yellow with elongate vertical white stripe with lateral dark brown margins extending to posterior margin of pronotum, at approximate median of pronotal length two additional vertical dark brown dashes on roughly parallel but removed from lateral margins, posterior of pronotum with diffuse darkening to pale brown adjacent to apical lateral margin above defined white line of posterior margin, mesoscutellum dark brown with two sets of whitish yellow markings adjacent to lateral angles, scutellum primarily whitish with dark brown M” shape medially; all coxae whitish beige with dark brown anterior margins on meso- and metacoxae, pro and mesofemora whitish yellow with dark brown spots forming diffuse vertical stripes on apical 1/2 to 2/3 length, metafemora primarily whitish yellow on ventral surface and dark brown on dorsal surface suffused with white spots, pro-tibiae and meso-tibiae white with faint pale brown spots, meta-tibiae whitish beige gradually darkening distally to pale brown adjacent tarsus and with short dark brown vertical stripe on ventral surface adjacent joint with femora as well as dark brown stripe ~ 1/5 of total length away from basal joint, all tarsal segments dark brown. ***Hemelytra***: predominantly whitish beige to yellowish with dark brown stripes and markings, clavus whitish beige becoming more yellow in coloration adjacent claval vein, claval vein contrasting golden brown, corium anteriorly white suffusing with dark brown spots adjacent median of clavus, distally becoming yellow-white and then dark brown at apex with membrane, corium also with dark vertical lines corresponding with claval suture, cubitus and radial veins, brief white patch adjacent apex of radial vein on interior margin, cuneus beige-white with dark brown checkerboard patterning on interior margin with membrane, membrane with dense brown maculae and two white patches along lateral margins adjacent cuneus apex and along medial edge, anal ridge pinkish yellow, membrane veins beige. ***Abdomen***: beige with defined horizontal black and white stripe along midline and diffuse dark brown horizontal streaks along dorsal and ventral surface, lateral tubercle beige, margin of genital segment thinly lined with dark brown to black.

**Surface and vestiture.** First antennal segment densely covered with whitish setae, dorsal surface of head, pronotum, mesoscutum, scutellum and hemelytra with long black erect setae mixed with silvery-sericeous setae distributed throughout as well as in defined areas such as patches of dark setae on apex of clavus, embolium, and interior margin of cuneus and white sericeous setae on medial stripe of mesoscutellum and scutellum, hemelytral surface texture otherwise matte; lateral margins of thorax and abdomen with distributed silvery sericeous setae.

**Structure. *Head***: tylus and clypeus strongly produced and visible in dorsal view, tylus nearly projecting anteriorly beyond clypeus, vertex relatively flat and slightly declining posteriorly, and dorsal surface of eyes surpassing vertex in lateral view, eyes relatively small with total heigh eye > ½ total height head in lateral view, interocular distance greater than both eye widths combined, antennal segment I length greater than width of head, apex of labium extending almost to genital capsule. ***Thorax***: collar relatively broad and well developed, pronotum elongate-trapezoidal in shape without well-developed calli and posterior margin relatively flat, mesoscutum and scutellum slightly rounded with declining margins towards clavus, legs narrow with hind legs especially elongate, pretarsus with convergent parempodia and developed pulvillus. ***Hemelytra***: lateral margins parallel sided, cuneus with shallow declivity. ***Genitalia***: Genital capsule with well-defined tubercle on left margin of gonopore (Fig. 3C). Endosoma with primary membranous relatively weakly developed and not extending far beyond median to basal margins of sclerotized processes; right sclerotized process elongated and narrow, forming almost L-shape, ending bluntly in relatively rounded apex; left sclerotized process also very elongate and relatively sinuous, nearly parallel in curvature to right process excluding apex, which straightens to overlap apex of right sclerite (Fig. 3D). Left paramere C-shaped with post-medial indentation on dorsal surface, broadening distally and terminating to small spine-like apex (Fig. 3F). Right paramere relatively narrow, thinning towards distal 1/5 until widening again into leaf-like apex with distal point (Fig. 3E).

**Female.** Similar to males in overall size and coloration, but with brachypterous wings whose apex barely surpasses median of abdomen length. Membrane reduced to sliver adjacent interior margins of cuneus (Fig. 3B).

**Measurements.** Table 1.

#### Hosts.

*Juniperusmonosperma* (Engelm.) Sarg. (Cupressaceae) and *Baileya* sp. (Asteraceae). Males also collected at a light sheet.

#### Distribution.

Jeff Davis Co., Texas and Catron Co. and Sandoval Co., New Mexico.

#### Etymology.

Named for Edward E. Riley, whose extensive fieldwork across Texas has led to the discovery of this species and many others. Noun in apposition.

#### Remarks.

This species clearly belongs in the Stonedahl (1988)*hopi* species group based on its possession of the following characters: the greyish brown coloration; the quadrate head with a prominent tylus; the small obovate eyes; the long first antennal segment; the narrow, black scalelike setae and silvery white, sericeous setae, and the endosoma with a reduced primary membranous sac of the endosoma with two large, sick-shaped sclerotized processes. *Phytocorisrileyi* sp. nov. appears to be most closely related to both *P.nigrolineatus* Knight and *P.pallidilineatus* Stonedahl based on the dark striped patterning on the pro-pleura, the coloration of the hemelytron, and the shape of the sclerites on the male endosoma. Both species are also found in Texas, but this species is separated from *P.nigrolineatus* by the yellow coloration of the claval vein versus the brown coloration of *P.nigrolineatus*, the fully developed black and white vertical lines on the first antennal segment (it is only a partial white line in *P.rileyi*, often not clearly defined), the differing shape of the right paramere (elongate in *P.rileyi*, squat and broad in *P.nigrolineatus*), and the shape of the left paramere (apex of paramere in *P.rileyi* is relatively expanded, whereas in *P.nigrolineatus* widest pre-apically). *Phytocorispallidilineatus* is also different from *P.rileyi* in the possession of two tubercles on the lateral margin of the gonopore versus the one tubercle found in *P.rileyi*, and the membrane between the sclerites in the male endosoma being more developed in *P.pallidilineatus*.

Only males were collected in Jeff Davis Co. and at lights, but subsequent investigation of undetermined *Phytocoris* material at the USNM revealed an additional single female and male specimen from New Mexico that included potential host plant information. As with other members of the *hopi* group, the female specimen is brachypterous with wings reduced to just the clavus and embolium. However, the overall coloration and patterning clearly matches the males, and we include this specimen’s information to round out our current knowledge of this species beyond Texas.

### ﻿ Phytocoris schmitzi sp. nov.

Taxon classificationAnimaliaHemipteraMiridae

2137B0EE-E75F-5207-AF0A-C9CA64A30A7C

https://zoobank.org/5F5BAB4F-2314-4B7C-A2D3-5876F1AA7090

Fig. 4

#### Type material.

***Holotype*** male, pinned. Original label: “United States: Texas: Jeff Davis Co., Davis Mts Preserve, Madera Canyon Overlook Trail, 30.6961 -104.1234, 5937 ft, 30.viii.2022 K. Menard, found on *Quercusgravesii* (Fagaceae)” (TAMU). ***Paratypes***: United States: Texas: Jeff Davis Co., Davis Mts. Resort, 5,800 ft (Marqua residence), UV, 30.62842°N, 104.08360°W, 4–5.vii. 2009, E & ML Riley, TAMU-ENTO X0615593 (UCONN) 1♂; same collecting event as holotype (USNM) 1♂, 1♀.

#### Diagnosis.

This species is diagnostic for the *rostratus* group based on the dark brown coloration, the subquadrate head with a broad vertex, a dorsum with narrow, dark, scalelike setae mixed with other types of vestiture, and male genitalia with greatly reduced primary membranous sac of endosoma with two sclerotized processes. It is unique, however, in the Z-like shape and spinose apex of the left endosomal sclerite in combination with the hook-like right endosomal sclerite.

**Figure 4.**
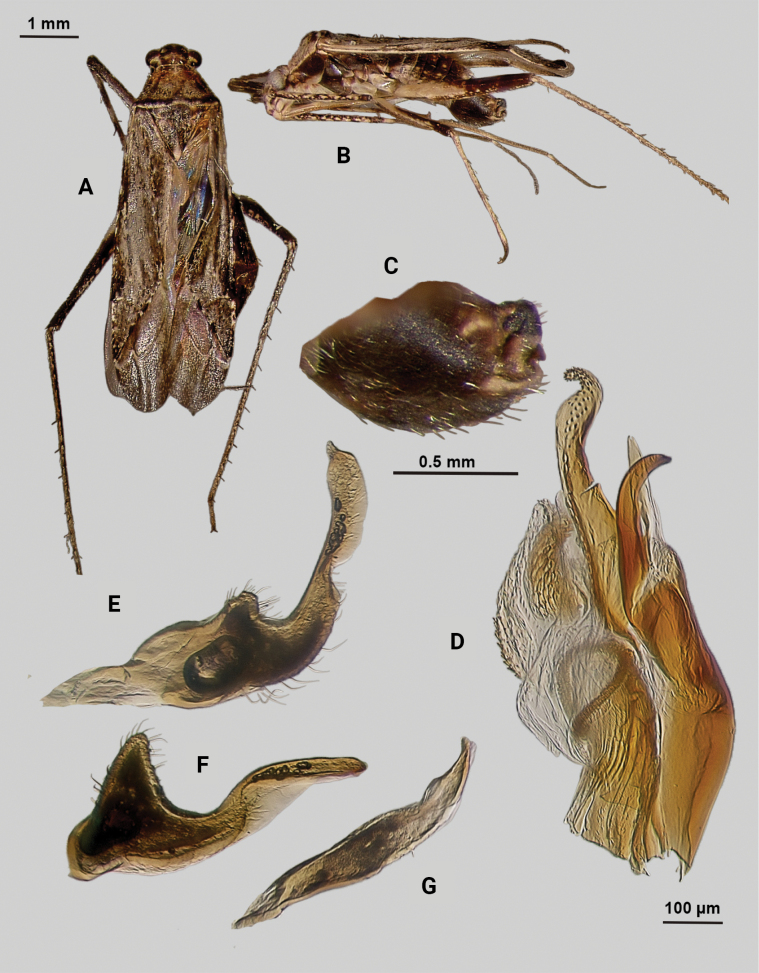
*Phytocorisschmitzi* sp. nov. **A** male dorsal habitus **B** male lateral view **C** male genital capsule in lateral view **D** endosoma **E** lateral view of left paramere **F** dorsal view of left paramere **G** lateral view of right paramere.

**Description. Male** (*n* = 2): Macropterous, medium-sized, lateral margins relatively straight. Total length clypeus to apex membrane from 4.5 mm, widest point across hemelytra 1.25 mm; general coloration dark brown and grey (Fig. 4A).

**Coloration. *Head***: predominantly pale brown in dorsal view, vertex with two small pale colored spots adjacent to eye margin and slightly darker horizontal line medially, frons with paler brown colored patterning associated with cyberium muscle attachment sites, clypeus dorsally pale with burgundy V-shaped pattern medially and parallel with antennal fossae and ventrally burgundy with two lateral white spots at apex, jugum white with dark burgundy horizontal band ventrad to antennal fossa in frontal view, maxillary plate and buccula burgundy-brown dorsally and ventrally white forming stripes that extend posteriorly, giving appearance of two horizontal dark stripes along head and continuing posteriorly behind eyes in lateral view, labrum pale white; labium basally pale white with first segment possessing short horizontal burgundy stripe that darkens distally to dark brown apex; antennal segment I dark burgundy-brown with pale maculae along dorsal surface, segment II pale brown with relatively narrow white band basally and slight darkening to darker brown on distal 1/3, segment III with narrow pale band basally and remainder color dark brown, segment IV completely dark brown; eyes brownish burgundy with some paler areas roughly corresponding to lateral stripe patterning on head. ***Thorax***: propleura dark brown with white on ventral margin with procoxa, mesopleura dark brown excluding white margins along mesocoxa, metapleura dark brown with ostiolar peritreme and metathoracic spiracle whitish yellow, pronotal collar dark brown with narrow vertical white stripe medially and two laterally posterior to eyes (Fig. 4B), anterior of pronotum pale brown with slightly darker brown calli and lateral margins, posterior pronotum primarily pale grey with irregular horizontal dark brown line parallel to white posterior margin, dark brown line on posterior margin possessing six slightly raised tumid points visible in lateral view, mesoscutum dark brown with spots of slightly paler coloration on lateral margins, scutellum primarily dark brown with slightly paler brown on posterior lateral margins and with white apex extending medially as white stripe to approximate median; pro and mesocoxa white with basal burgundy-brown spot, metacoxa white with basal 1/3 burgundy-brown and with short basal-posterior burgundy-stripe distally, pro and mesofemora pale whitish yellow basally with increasing coverage of burgundy-brown maculae distally that sometimes form vertical bands, metafemora with very dense burgundy-brown maculae with some white areas on ventral surface basally, protibiae dark brown with white maculae on basal 0/3 and one white stripe medially and second at ~ 1/4 length distally, mesotibia with similar patterning to protibiae but with less defined margins of white stripes, metatibiae primarily pale brown with dark brown maculae basally becoming more diffuse distally, all tarsal segments dark brown. ***Hemelytra***: predominantly pale grey with darker blackish brown diffuse areas, clavus darker brown with sporadic dark brown maculae for majority of anterior surface and transitioning to pale grey along claval margin and claval suture, corium pale brownish grey darkening to dark brown to form lines along costal vein, costal margins and lateral margins adjacent to cuneal fracture as well as whitish patches adjacent to anterior margin of cuneus extending anteriorly approximately equidistant to length of cuneus, corium also with black spot corresponding with dark setae on distal margin with cuneus, cuneus primarily pale brown with thin white margin along cuneal fracture and dark brown to black patches at apex and median margin with membrane corresponding to patches of black setae, membrane primarily comprised of brown maculae excluding two white patches along lateral margins adjacent cuneus apex, veins brown to dark brown. ***Abdomen***: burgundy-brown coloration dorsally and distally with whiteish pale on ventral surface of anterior 1/2, apex of lateral tubercle and distal margins of genital segment transitioning to greyish white coloration.

**Surface and vestiture.** Dorsal surface of head, pronotum, mesoscutum, scutellum, and hemelytra with long black erect setae evenly distributed and mixed with silvery-sericeous setae as well as in defined patches such as the apex of the clavus, distal apex of the corial margin, and two patches adjacent margin of corium and cuneus with the wing membrane, hemelytral surface texture otherwise matte. **Structure: *Head***: clypeus visible in dorsal view, vertex rounded and slightly declining posteriorly, dorsal surface of eyes not surpassing vertex in lateral view, eye height ~ 1/2 of total height of head in lateral view, interocular distance slightly < 1/2 total width head, antennal segment I relatively long with length nearly twice width head, apex of labium extending past hind coxae. ***Thorax***: collar well-developed, pronotum trapezoidal in shape with faintly developed calli, posterior of pronotum with six weakly protruding tumid points adjacent posterior margin, posterior margin weakly concave in dorsal view, mesoscutum and scutellum relatively flat with declining margins towards clavus, legs relatively narrow with hind legs elongate, pretarsi with convergent parempodia and developed pulvillus. ***Hemelytra***: lateral margins nearly parallel-sided, cuneus with weak declivity. ***Abdomen***: less than ½ of total body length, apex not reaching apex of wing membrane, genital capsule relatively large, nearly 1/2 total length of abdomen. ***Genitalia***: Genital capsule with relatively small but well-developed tubercle on left margin, pointed at apex (Fig. 4C). Endosoma with broad membranous lobes covered with minute spines and spiculi, and three medially located well-developed sclerites; right sclerite basally rounded, distally narrowed with hook-like apex, medial narrow and elongate as well as sclerite weakly sclerotized, and left sclerite elongate apically covered with spines and forming sinuate Z-shape (Fig. 4D). Left paramere L-shaped with relatively broad dorsal margin medially marked with dorsal notch and pointed apex along with pointed sensory lobe (Fig. 4E, F). Right paramere elongate and narrow, with slight preapical widening before tapering into a hook-like apex (Fig. 4G).

**Female.** Similar to male in coloration and structure, but slightly larger in size.

**Measurements.** Table 1.

#### Hosts.

*Quercusgravesii* Sudworth (Fagacaeae). One additional male was collected at lights.

#### Distribution.

Davis Mountains, Jeff Davis County, Texas.

#### Etymology.

Named for coleopterist Ashley Schmitz whose invitation to assist in the biodiversity survey of the Nature Conservancy property led to the discovery of this new species. Noun in apposition.

#### Remarks.

Two males and one female specimen was available for dissection and documentation of this new species; both males were dissected to confirm their species identity. One additional male was collected with this series but was teneral and therefore not measured or dissected. *Phytocorisschmitzi* also fits into the *rostratus* group of Stonedahl (1988) based on the same characters that place *P.marqua* in the category, particularly the characteristics of the two sclerotized processes of the male genitalia. *P.schmitzi* is most superficially similar to *Phytocoriscienega* from Presidio, Texas in the coloration patterning and possession of 4–6 weakly elevated points on the posterior margin of the pronotum. However, the first antennal segment in *P.schmitzi* is wider than the width of the head unlike narrower in *P.cienega*. Also, there were significant differences in the male genitalia including the relatively wide and developed tubercle on the lateral margin of the genital capsule and the sinuate Z-like shape of the left sclerotized process (Fig. 4D).

##### ﻿Known species of *Phytocoris* Fallén recorded from Jeff Davis County, Texas

Literature references: 1. Stonedahl (1988).

### ﻿ Phytocoris baboquivari

Taxon classificationAnimaliaHemipteraMiridae

Stonedahl, 1988 (rostratus species group). 1

6101697C-7F7B-5546-8D5B-F6F523C27CEB

Fig. 5

#### Specimens examined.

Davis Mountains Nature Preserve, McIvor Center. 30.6986, -104.1161, 5895 ft, 29.vii.2022, K Menard, found on *Juniperusdeppeana* (Cupressaceae) (UCONN), 1♂; Davis Mountains, Madera Canyon Road, Sweep. 30.6986, -104.1161, 5895 ft, 28.vii.2022, A Schmitz (UCONN), 1♂; Ft. Davis, 30.v.1959, Howden & Becker, (CNC) AMNH_PBI 00419791, 1♂; Ft. Davis, 30.v.1959, Howden & Becker, (CNC) AMNH_PBI 00419799, 1♀; Wild Rose Pass on Hwy. 17, 7.viii.1992, W Godwin & E Riley, X0961678 (TAMU), 1♀.

**Figure 5.**
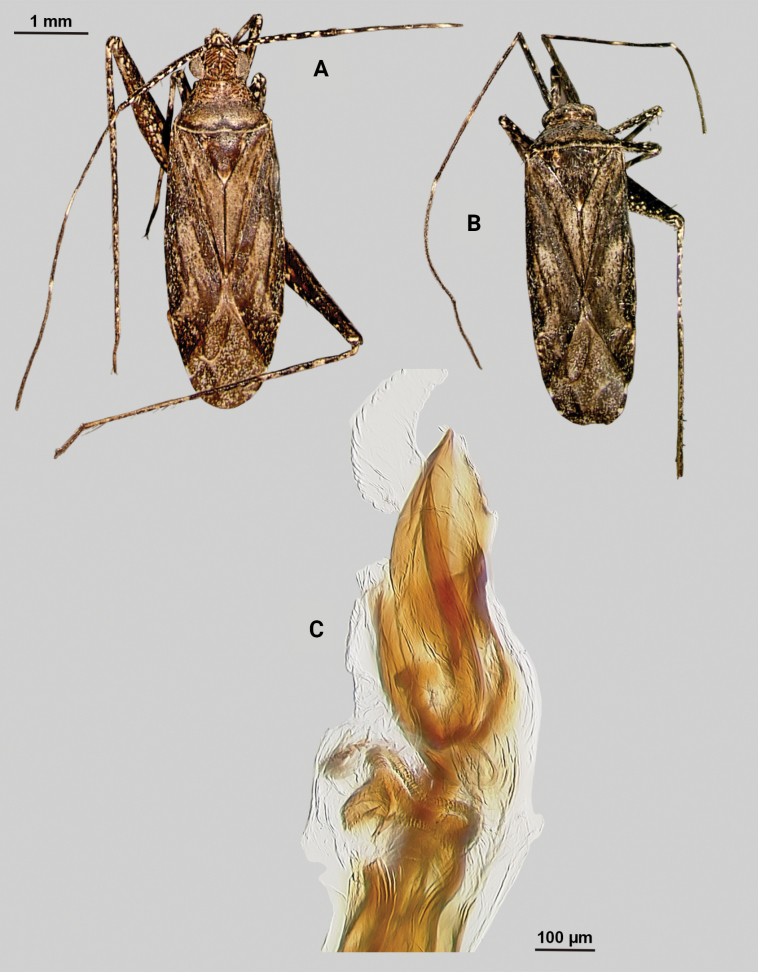
*Phytocorisbaboquivari***A** male dorsal habitus **B** female dorsal habitus **C** endosoma.

### ﻿ Phytocoris berbericola

Taxon classificationAnimaliaHemipteraMiridae

Stonedahl, 1988 (aurora species group). 1

36B83E96-D812-5D40-83C6-C9C9A040EC25

Fig. 6

#### Specimens examined.

Davis Mountains State Park, 10.xix.1988, RS Anderson (TAMU), X0426919, 1♀; Ft. Davis, 30.v.1959, Howden & Becker, (CNC) AMNH_PBI 00419801, 1♀.

**Figure 6.**
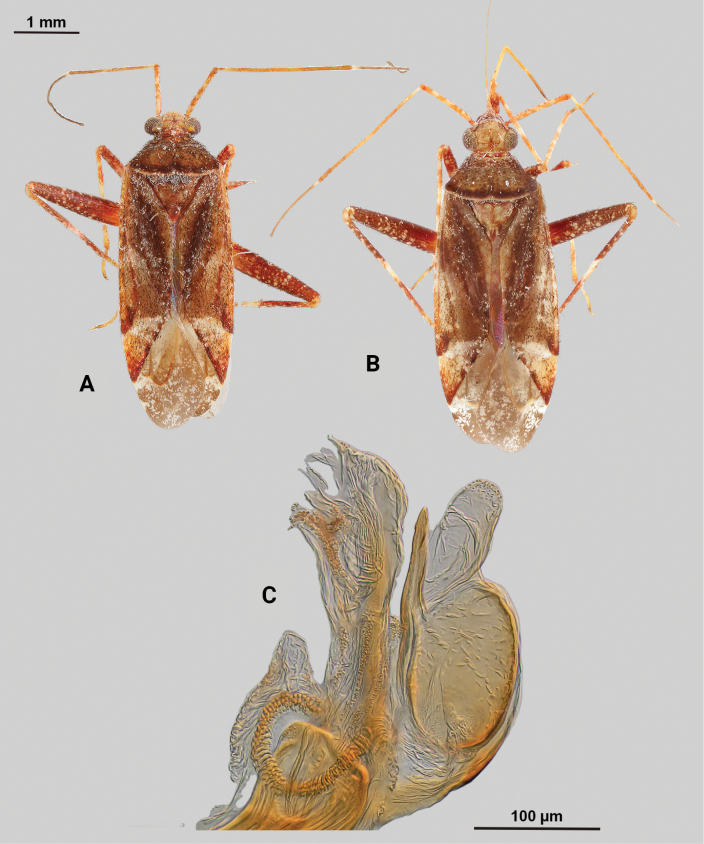
*Phytocorisberbericola***A** male dorsal habitus **B** female dorsal habitus **C** endosoma.

### ﻿ Phytocoris carnosulus

Taxon classificationAnimaliaHemipteraMiridae

Van Duzee, 1920 (carnosulus species group). 1

865B4131-726C-5351-A9B6-CF113C534020

Fig. 7

#### Specimens examined.

Davis Mountains Nature Preserve, McIvor Center, 30.6986, -104.1161, 5895 ft. 29.vii.2022, K Menard, (UCONN), 1♂; Davis Mountains Resort, upper Limpia Creek Canyon, 6180 ft, 30°37'48"N, 104°07'59"W, 14.iv.2002, UV light, EG Riley & MJ Yoder, (TAMU), X0896969, 1♂; Ft. Davis, 30.v.1959, Howden & Becker, (CNC) AMNH_PBI 00419806, 1♂; Davis Mt., Ft. Davis, 11.viii.1957, RH Arnett, (CNC) AMNH_PBI 00419807–00419808, 2♂; Davis Mountains State Park, 11iv.2002, blacklight, Cognato & Usener, (TAMU) X0897241, 1♂; Davis Mountains Resort, Marqua residence, UV light, 5800 ft, 30.62842°N, 104.0836°W, 4–5.vii.2009, E & ML Riley, (TAMU), X06157159, 1♂, X0613939,1♂; 11.vii.2002, UV light, EG Riley & MJ Yoder, (TAMU), X0892242, 1♂, X0893204, 1♂, X0892058, 1♂, X0893239, 1♂, X0894672, 1♂.

**Figure 7.**
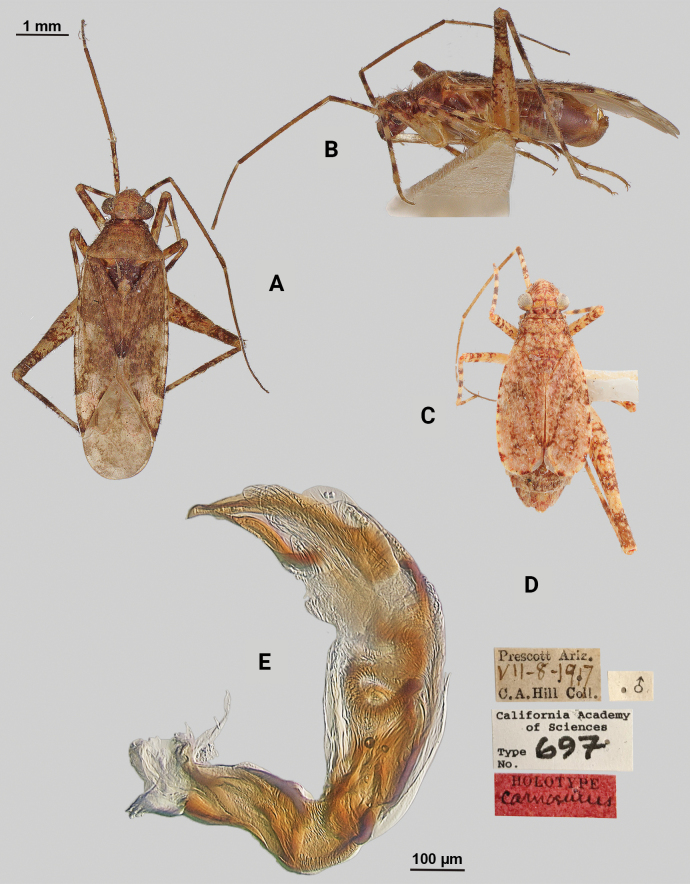
*Phytocoriscarnosulus***A** holotype male dorsal habitus **B** holotype male lateral view **C** female dorsal habitus **D** holotype specimen labels **E** female dorsal habitus **F** endosoma.

### ﻿ Phytocoris cinereus

Taxon classificationAnimaliaHemipteraMiridae

Stonedahl, 1988 (hopi species group). 1

D4659692-CBE3-5CF6-A4B4-E1E3F8B5748B

Fig. 8

#### Specimens examined.

Davis Mountains State Park, V-25-2001, J Usener, MV (TAMU), 894662 1♂. Davis Mountains State Park, 30.59583°N, 103.92972°W, 1533 m, 6.x.1988, MD Schwartz, *Berberistrifoliolata* Moric. (Berberidaceae), (AMNH_ENT 00071419) 2♀.

**Figure 8.**
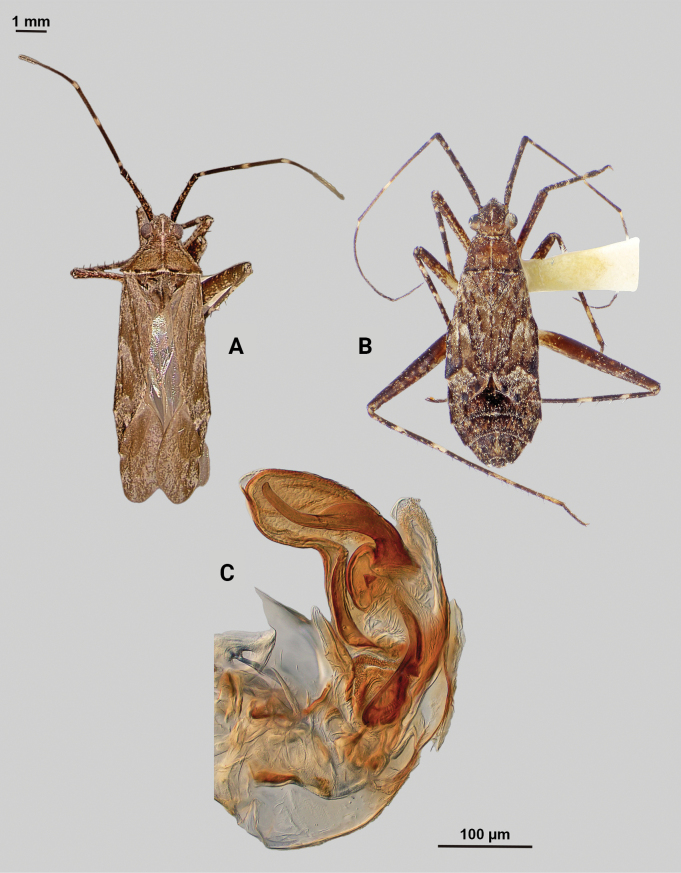
*Phytocoriscinereus***A** male dorsal habitus. **B** female dorsal habitus. **C** endosoma.

### ﻿ Phytocoris decurvatus

Taxon classificationAnimaliaHemipteraMiridae

Knight, 1968 (junceus species group). 1

ACD8D8C3-1238-5B22-87E0-33306C90396F

Fig. 9

#### Specimens examined.

Davis Mountains Resort, Marqua residence, 5800 ft, 30.62842°N, 104.08360°W, UV light, 14.x.2011, EG Riley, (TAMU), X0893771 1♂, X0892613 1♂, X0894139 1♂, X0894420 1♂; X0892719 1♀, X0893622 1♀, X094005 1♀, X0894312 1♀, X089455 1♀, X0891699 1♂, X0893079 1♂; 4–5.vii.2009, E & ML Riley, UV light (TAMU), X0613984 1♂, X0615821 1♂, X0615512 1♂, X0615736 1♂, X0614854 1♂, X0613977 1♂, X0615796 1♂; 8.vii.1992, W Godwin & E Riley, (TAMU), XO904452 1♂; Rest stop 9.5 mi S jct. highway 118 & 106, 24.vii.1989, EG Riley, (TAMU), X0904452 1♂; Davis Mountains Nature Conservancy, McIvor Center, 30.6986, -104.1161, 5895 ft, MV light, K Menard, (UCONN), 1♂.

**Figure 9.**
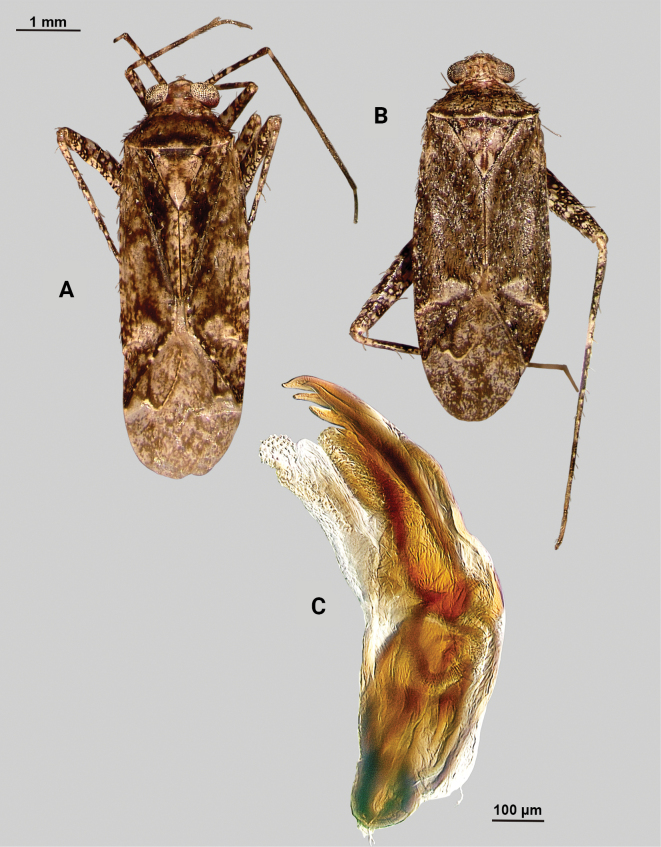
*Phytocorisdecurvatus***A** male dorsal habitus **B** female dorsal habitus **C** endosoma.

### ﻿ Phytocoris interspersus

Taxon classificationAnimaliaHemipteraMiridae

Uhler, 1895 (interspersus species group).

037698F5-879C-5E12-A27B-7077C6DFD189

Fig. 10

#### Specimens examined.

Davis Mountains Preserve, Madera Canyon Overlook Trail, 30.6961, -104.1234, 5937 ft, 30.viii.2022, K Menard, (UCONN), 1♀; McDonald Observatory, 3.ix.1986, DA East, PW Kovarik, Haack, (TAMU), X0889398 1♀, X0889295 1♀, X0890194 1♂, X0890200 1♀, X0889440 1♀, X0889009 1♀, X0889683 1♀, X0887760 1♂; Davis Mountains State Park, 5200 ft, 6.x.1988, MD Schwartz, *Quercuschihuahuaensis* Trel. (Fagaceae), Det. K Nixon (LHB Hort), (AMNH), AMNH_ENT_00074543 1♂, AMNH_ENT_00074574 1♀.

**Figure 10.**
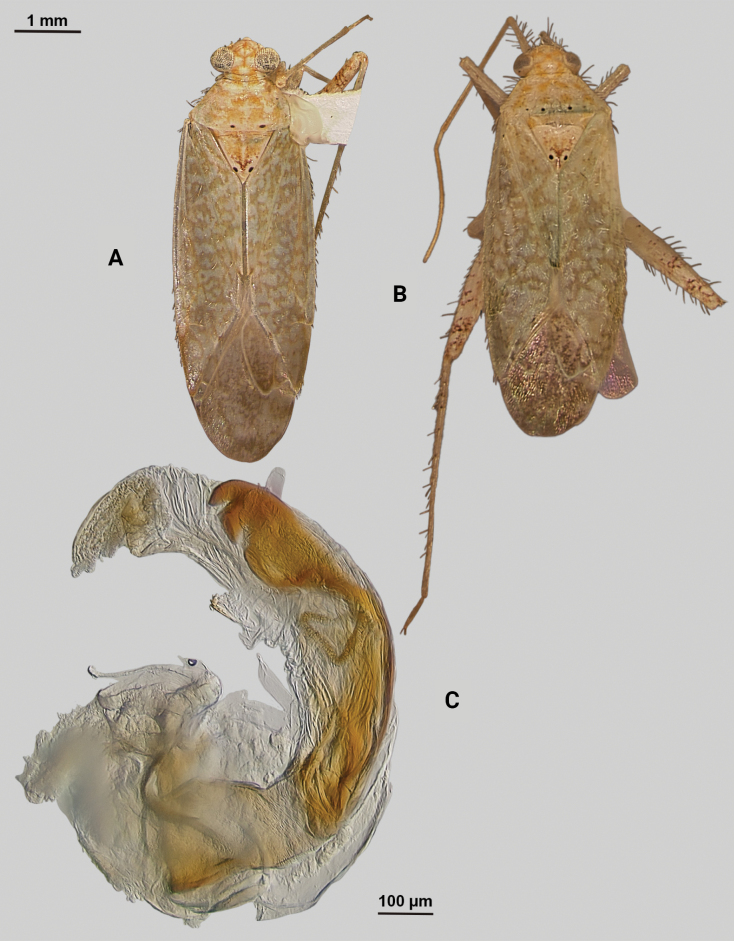
*Phytocorisinterspersus***A** male dorsal habitus **B** female dorsal habitus **C** endosoma.

### ﻿ Phytocoris juniperanus

Taxon classificationAnimaliaHemipteraMiridae

Knight, 1968 (juniperanus species group). 1

7359C26C-32B0-5A3B-999B-496FD37FC1AF

Fig. 11

#### Specimens examined.

Davis Mountains Resort, D. Marqua residence, 5800 ft, 8.vii.1992, W Godwin & E Riley, (TAMU), X0904823 1♂; Davis Mountains Hwy 118, McDonald Observatory, 30°41'12"N, 104°02'04"W, 1892 m, 14.viii.2001, JC Schaffner, (TAMU), X0965350 1♂.

**Figure 11.**
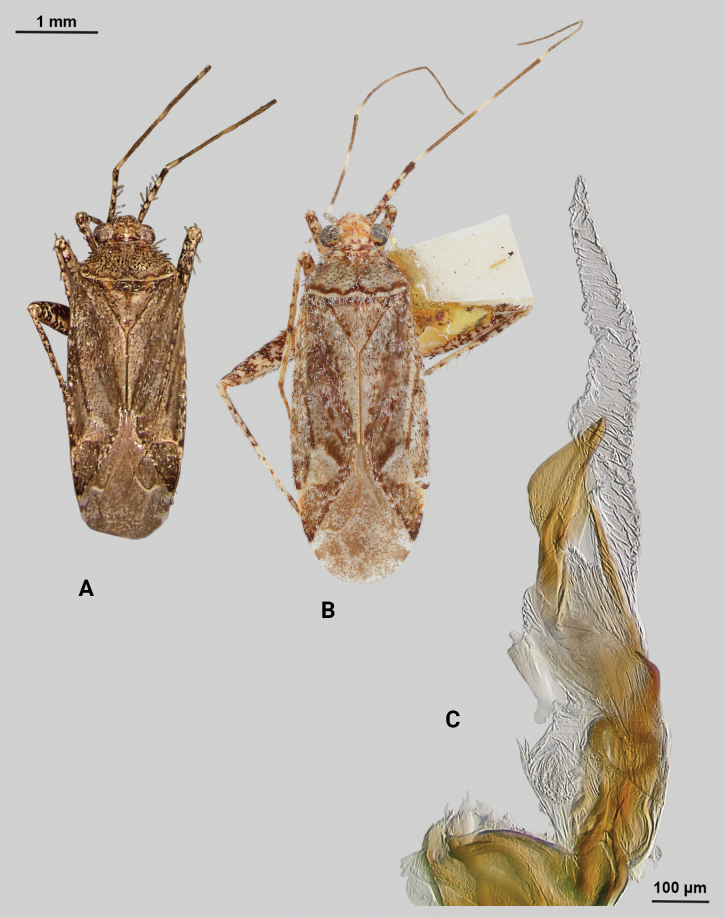
*Phytocorisjuniperanus***A** male dorsal habitus **B** female dorsal habitus **C** endosoma.

### ﻿ Phytocoris listi

Taxon classificationAnimaliaHemipteraMiridae

Knight, 1928 (listi species group). 1

AAAF4E0F-3911-5652-8B7F-5B40BF7E230B

Fig. 12

#### Specimens examined.

Fort Davis, 9.viii.1969, VV Board & JE Hafernik, (TAMU), X0892919 1♂; Sawtooth Mountain Park, 29.v.1973, GC Gaumer & WE Clark, (TAMU), X0890480 1♂.

**Figure 12.**
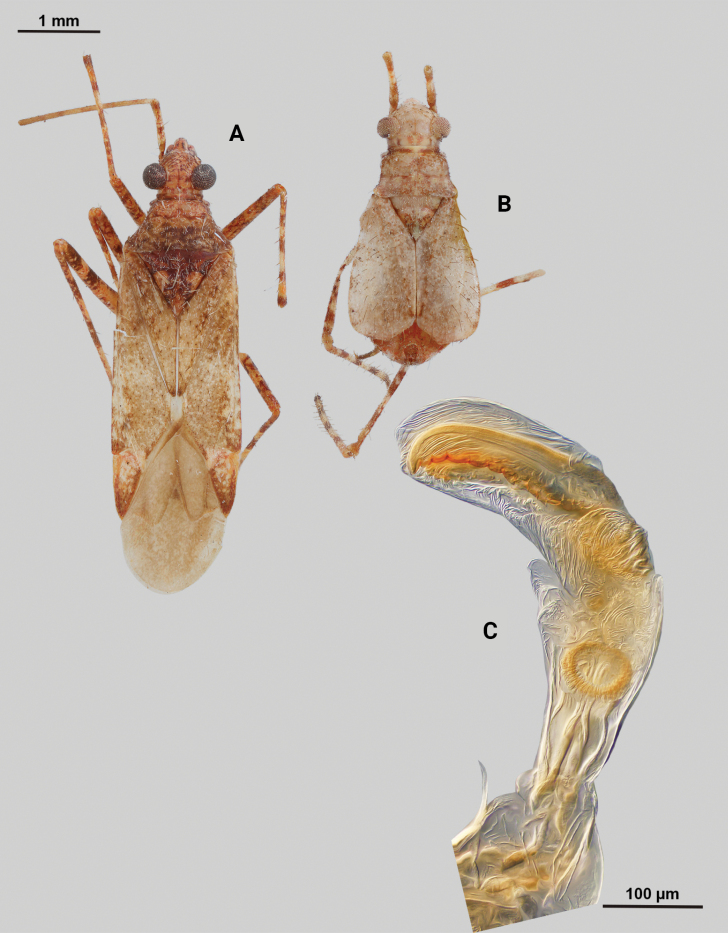
*Phytocorislisti***A** male dorsal habitus **B** female dorsal habitus **C** endosoma.

### ﻿ Phytocoris maricopae

Taxon classificationAnimaliaHemipteraMiridae

Stonedahl, 1988 (rostratus species group). 1

5251E8CC-09B6-554D-A1A4-D8F49935CC69

Fig. 13

#### Additional specimens.

Texas: Presidio Co.: Presidio, 29.56056°N, 104.37167°W, 788 m, 21.viii.1968, JE Hafernik, (CNC) (AMNH_PBI 00419812) 1♂.

#### Notes.

This species was recorded from the literature in Jeff Davis County by a paratype designated by Stonedahl deposited at the CAS. For documentation purposes of the habitus and male genitalia of this species, we utilized specimens from the synoptic set deposited at the CNC instead to minimize potential damage to the one type specimen for the purpose of this paper.

**Figure 13.**
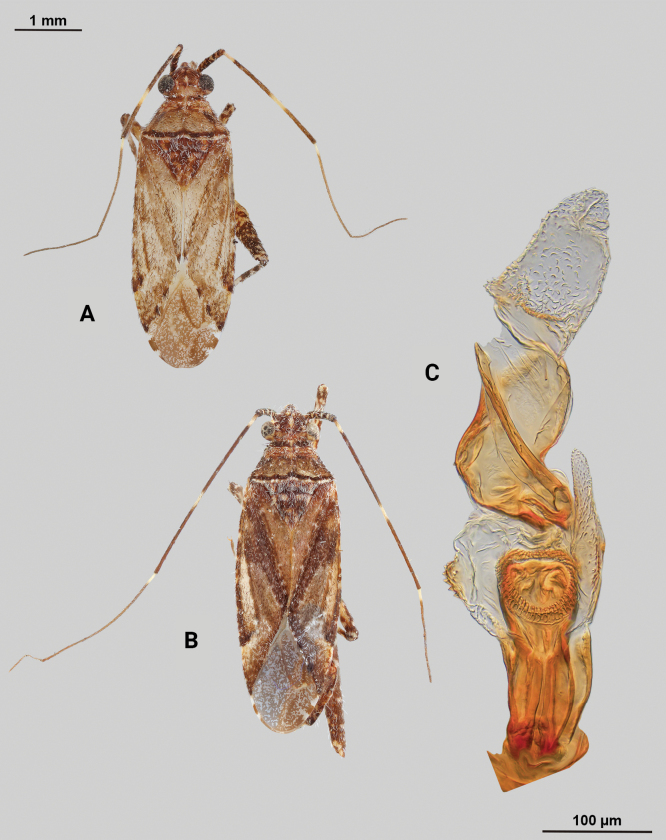
*Phytocorismaricopae***A** male dorsal habitus **B** female dorsal habitus **C** endosoma.

### ﻿ Phytocoris olseni

Taxon classificationAnimaliaHemipteraMiridae

Knight, 1923 (pulchellus species group). 1

F9372B59-DAAB-5CB1-907D-CBF5461F0F73

Fig. 14

#### Specimens examined.

Davis Mountains Nature Conservancy, McIvor Center, 30.6986, -104.1161, 5895 ft, 29.viii.2022, K Menard, *Quercusemoryii* (Fagaceae) (UCONN), 1♂ 2♀; UV light (UCONN), 1♂ 1♀; Davis Mountains Resort, Marqua residence, 5800 ft, 30.62842°N, 104.08360°W, 4–5.vii.2009, E & ML Riley, UV light (TAMU), X0614291 1♂, X0614128 1♂; X0615700 1♂; 14.x.2011, EG Riley, UV light (TAMU), X0892569 1♂; 17.v.1993, D Marqua, Malaise trap (TAMU), X0963590 1♀, X0619262 1♂.

**Figure 14.**
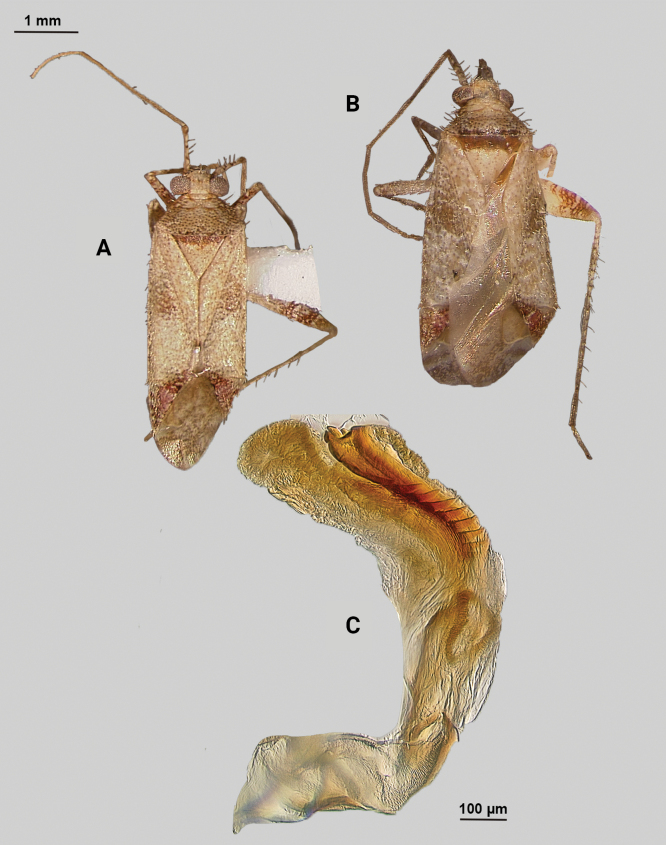
*Phytocorisolseni***A** male dorsal habitus **B** female dorsal habitus **C** endosoma.

### ﻿ Phytocoris ramosus

Taxon classificationAnimaliaHemipteraMiridae

Uhler, 1897 (conspurcatus species group).

1E6E90B5-3D78-5D90-8C0F-EB17DC03F104

Fig. 15

#### Specimens examined.

Davis Mountains, Madera Canyon, 26.v.2001, J Usener, MV (TAMU), X0895017 1♀, X0895438 1♀.

**Figure 15.**
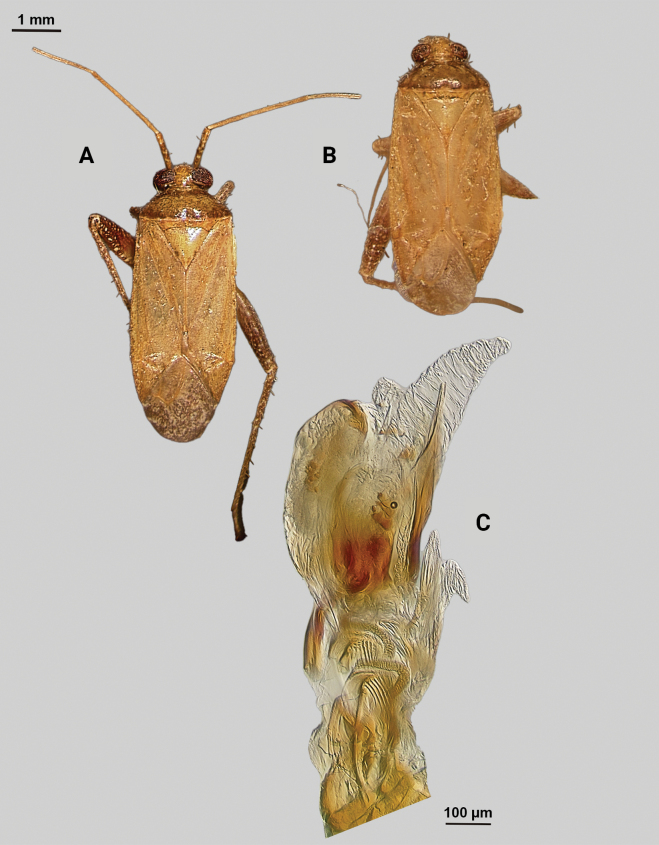
*Phytocorisramosus***A** male dorsal habitus **B** female dorsal habitus **C** endosoma.

#### Additional specimens.

Presidio Co.: 29.vii.1968, ML Allender, (TAMU), X0962477 1♂.

#### Note.

Because only females were available for records in Jeff Davis County, male habitus and genitalia photos were taken from specimens collected in adjacent Presidio County.

### ﻿ Phytocoris relativus

Taxon classificationAnimaliaHemipteraMiridae

Knight, 1968 (conspurcatus species group).

B3CA7C25-0021-586C-B053-0989D6A9878B

Fig. 16

#### Specimens examined.

Fort Davis State Park, 24.viii.1970, JR Sweet & MH Sweet (TAMU), X0957617 1♀, X0889901 1♂, X0958831 1♂, X0959031 1♂; 1 mi W of Fort Davis, 20.vii.1968 (TAMU), X0959886 1♀; 22.viii.1968 (TAMU), X0889812 1♂; 5 mi S of Fort Davis, 6.vi.1972, WE Clark, (TAMU), X0891178 1♂.

**Figure 16.**
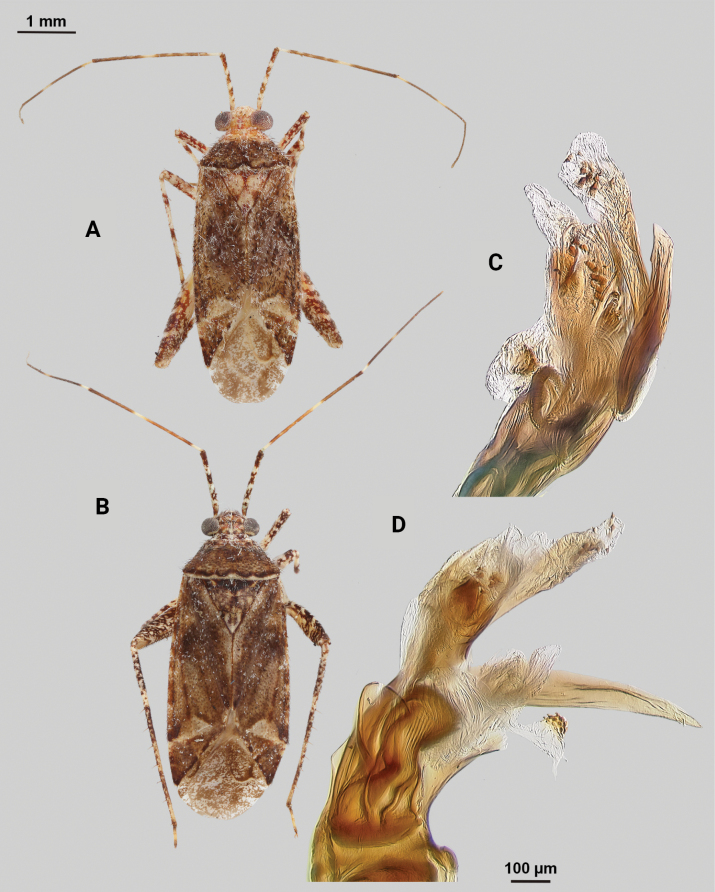
*Phytocorisrelativus***A** male dorsal habitus **B** female dorsal habitus **C** endosoma anterior view **D** endosoma posterior view.

### ﻿ Phytocoris roseipennis

Taxon classificationAnimaliaHemipteraMiridae

Knight, 1934 (roseipennis species group). 1

99F8995D-60B6-5063-8D24-418BF4FF1492

Fig. 17

#### Specimens examined.

Davis Mountains, Madera Canyon Road 0.5 mi S water pump, 30.6649, -104.1544, 6216 ft, 29.viii.2022, K Menard, found on Poaceae sweeping (UCONN), 1♂; Davis Mountains, Madera Canyon Road, sweeping, 30.6686, -104.1161, 5895 ft, 28.viii.2022 A Schmitz, (UCONN), 1♂ 2♀; Davis Mountains Nature Conservancy, McIvor Center, 30.6986, -104.1161, 5895 ft, 29.vii.2022, K Menard (UCONN), 1♀.

**Figure 17.**
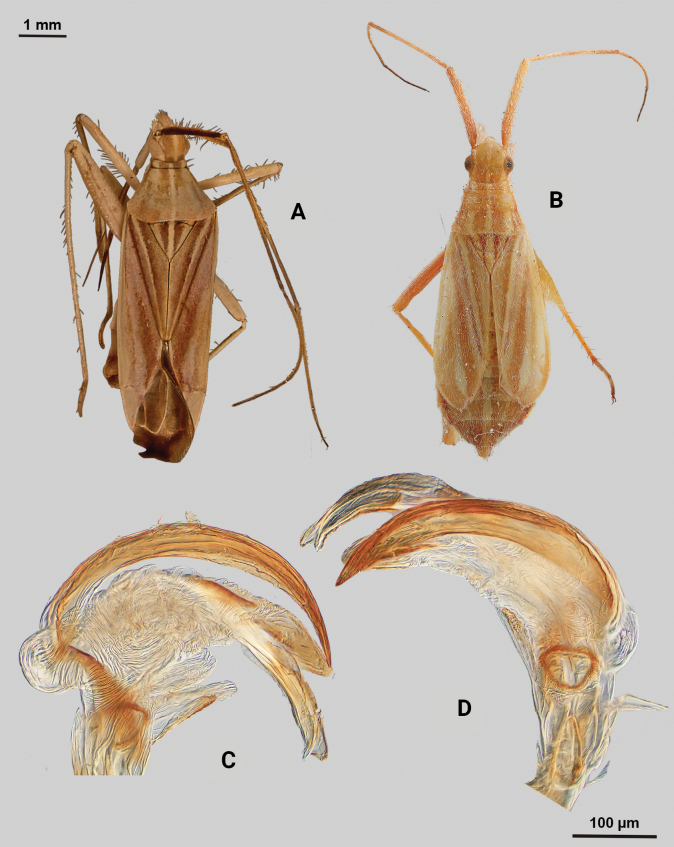
*Phytocorisroseipennis***A** male dorsal habitus **B** female dorsal habitus **C** endosoma posterior view **D** endosoma anterior view.

### ﻿ Phytocoris schuhi

Taxon classificationAnimaliaHemipteraMiridae

Stonedahl, 1988 (fraterculus species group).

697C5E2F-05A5-5F08-9C19-3B1C2F400B9B

Fig. 18

#### Specimens examined.

McDonald Observatory, 10.ix.1988, RS Anderson, (TAMU), X0894515 1♀, X0893339 1♂, X0891748 1♀.

**Figure 18.**
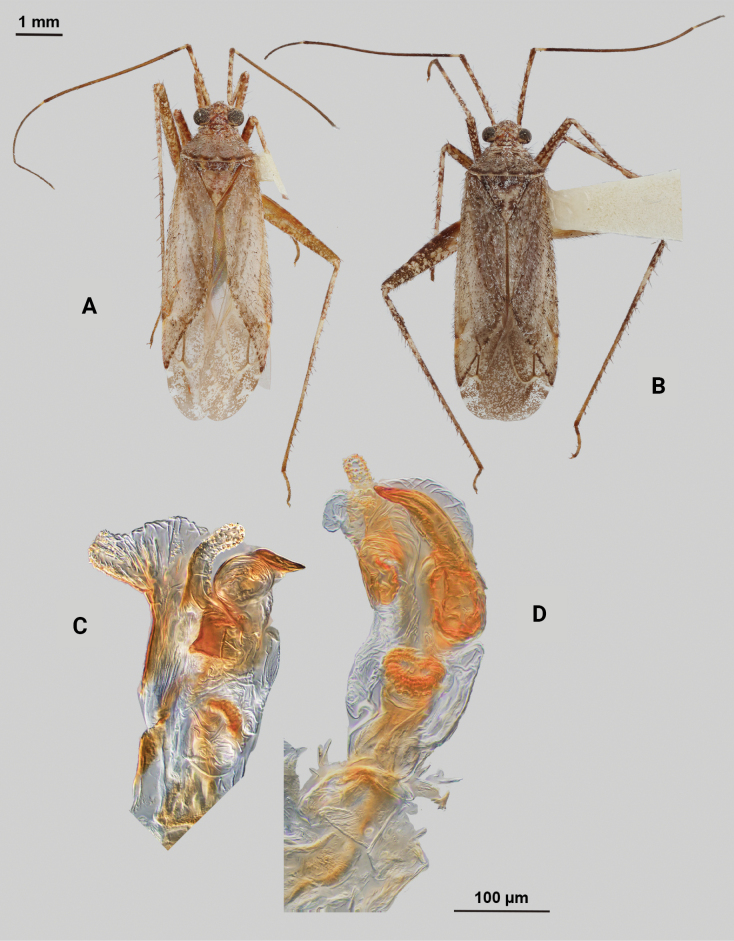
*Phytocorisschuhi***A** male dorsal habitus **B** female dorsal habitus **C** endosoma posterior view **D** endosoma anterior view.

### ﻿ Phytocoris seminotatus

Taxon classificationAnimaliaHemipteraMiridae

Knight, 1934 (plenus species group), new state record

F757A9C9-6A45-542D-B24F-A02CBA6E6B7F

Fig. 19

#### Specimen examined.

Davis Mountains Resort, 5800 ft, 24.ix.2005, DG Marqua, (TAMU), X0896445, 1♂.

#### Additional specimens.

Brewster Co.: Big Bend National Park, Canyon Camp Area no. 4,4700 ft, 29°15'59"N, 103°14'04"W, 1.x.2005, Raber & Riley, 57 (TAMU), X096259 1♀, X0963758 1♂; Presidio Co.: Big Bend Ranch State National Area, Tres Papalotes, 1.xi.1989, 89/027, G Zolnerowich, (TAMU), X0961740 1♀.

**Figure 19.**
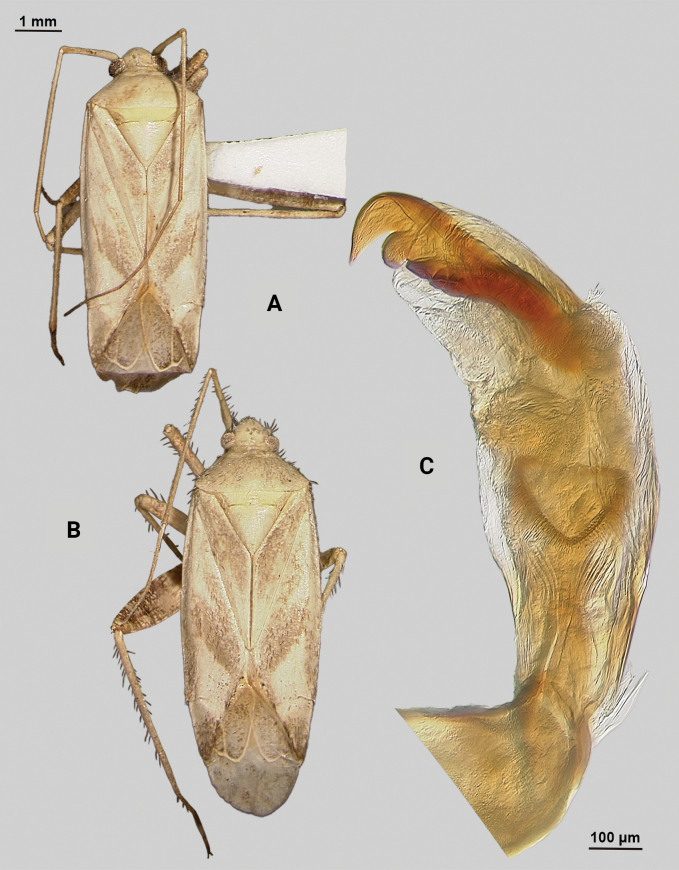
*Phytocorisseminotatus***A** male dorsal habitus **B** female dorsal habitus **C** endosoma.

#### Note.

Habitus figures are from adjacent Brewster Co., Texas specimens because the lone male specimen of *P.seminotatus* from Jeff Davis Co. was directly pinned, and unfortunately broke in half when the genitalic capsule was being dissected. The component body parts were re-glued onto a card and associated with the genitalia, which was imaged for the manuscript.

### ﻿ Phytocoris simulatus

Taxon classificationAnimaliaHemipteraMiridae

Knight, 1928 (fraterculus species group). 1

367FDFDA-5713-5E57-9CED-82CA562CF33F

Fig. 20

#### Specimens examined.

Davis Mountain Nature Preserve, McIvor Conservation Center, 30.695554, -104.121378, 5902 ft, 17.v.2022 K Menard, UV light (UCONN), 1♂ 1♀; TNC Davis Mountains Preserve, Right Hand Loop, 30.837361, -104.0338989, 17.v.2021, AJ Schmitz, (UCONN), 1♂; Davis Mountains Nature Preserve, 0.25 mi S. McIvor Conservation Center, 30.699739, -104.115493, 5903 ft, 18.v.2022, K Menard, found on *Pinuscembroides* (Pinaceae) (UCONN), 4♂ 2♀; Davis Mountains Hwy 118, 8 mi NW McDonald Observatory, 30°42'22"N, 104°06'17"W, 1892 m, 14.viii.2001, JC Schaffner, (TAMU), X0964873 1♀, X0965043 1♀, X0965087 1♂, X0903693 1♂, X0964010 1♂, X0964011 1♀, X0966544 1♂, X0964749 1♂, X0904140 1♀; Davis Mountains Resort, Marqua residence, 5800 ft, 30.62842°N, 104.08360°W, 21.vi.2011, Quinn & Riley, (TAMU), X0619885 1♂, X0620181 1♂, X0620533 1♂, X0620265 1♂, X0620422 1♂, X0620491 1♂; VIII-8-1992, UV light, W Godwin & E Riley, (TAMU), X0962470 1♂, X0962855 1♂, X0962417 1♂; 31.v.1993, Malaise Trap, D Marqua, (TAMU), X0966647 1♂; 10–17.v.1993, Malaise Trap, D Marqua, (TAMU), X0968119 1♂; 14.x.2011, UV light, EG Riley, (TAMU), X0890293 1♀, X0894660 1♂, X0891329 1♂, X0891883 1♀; Rest Stop, 9.5 mi S junction Hwy 118 on 166, 9.viii.1992, UV light, W Godwin & E Riley, (TAMU), X0901968 1♂, X0903350 1♂; Madera Canyon rest stop, highway 118, 9.vii.1992, W Godwin & E Riley, (TAMU), X0968946 1♂.

**Figure 20.**
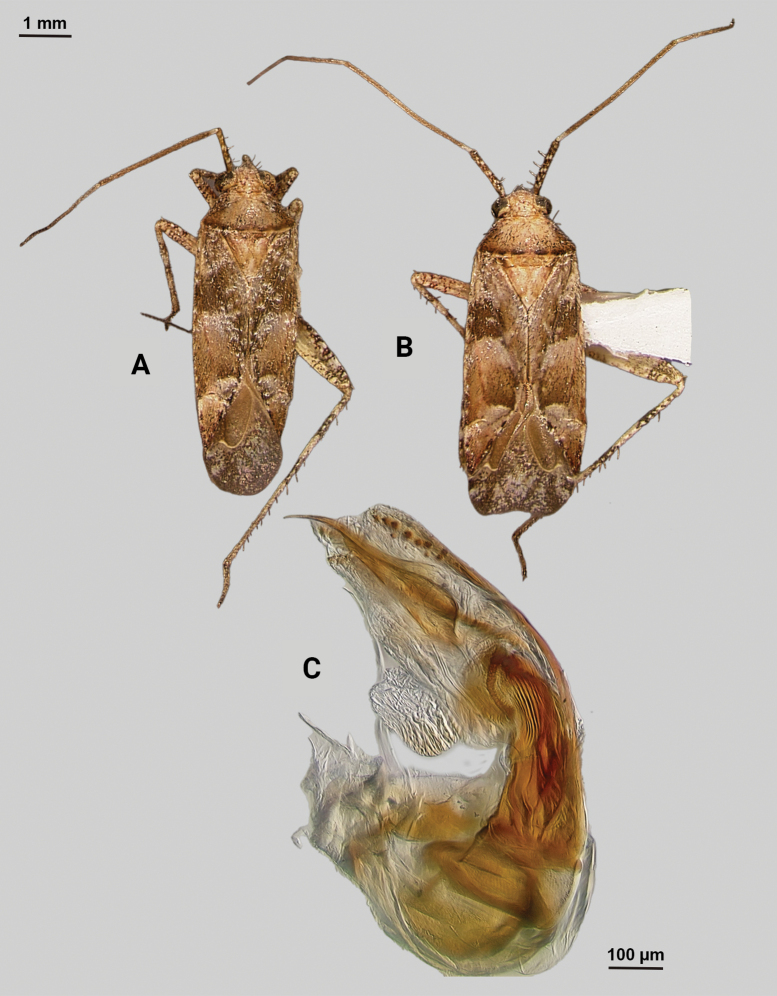
*Phytocorissimulatus***A** male dorsal habitus **B** female dorsal habitus **C** endosoma.

### ﻿ Phytocoris vanduzeei

Taxon classificationAnimaliaHemipteraMiridae

Reuter, 1912 (juniperanus species group). 1

00E76098-1E2B-52BD-BB4D-187F6F4BA98C

Fig. 21

#### Specimens examined.

Arizona: Cochise County, vicinity of Portal, 2.v.1978, RT Schuh, collected on *Larreadivaricata*, (CNC) AMNH_PBI_419933, 1♂; New Mexico: Eddy County, Hope, 22.vii.1967, LA Kelton, collected on *Larrea*sp. (CNC) AMNH_PBI_419921, 1♀; AMNH_PBI_419920, 1♂.

**Figure 21.**
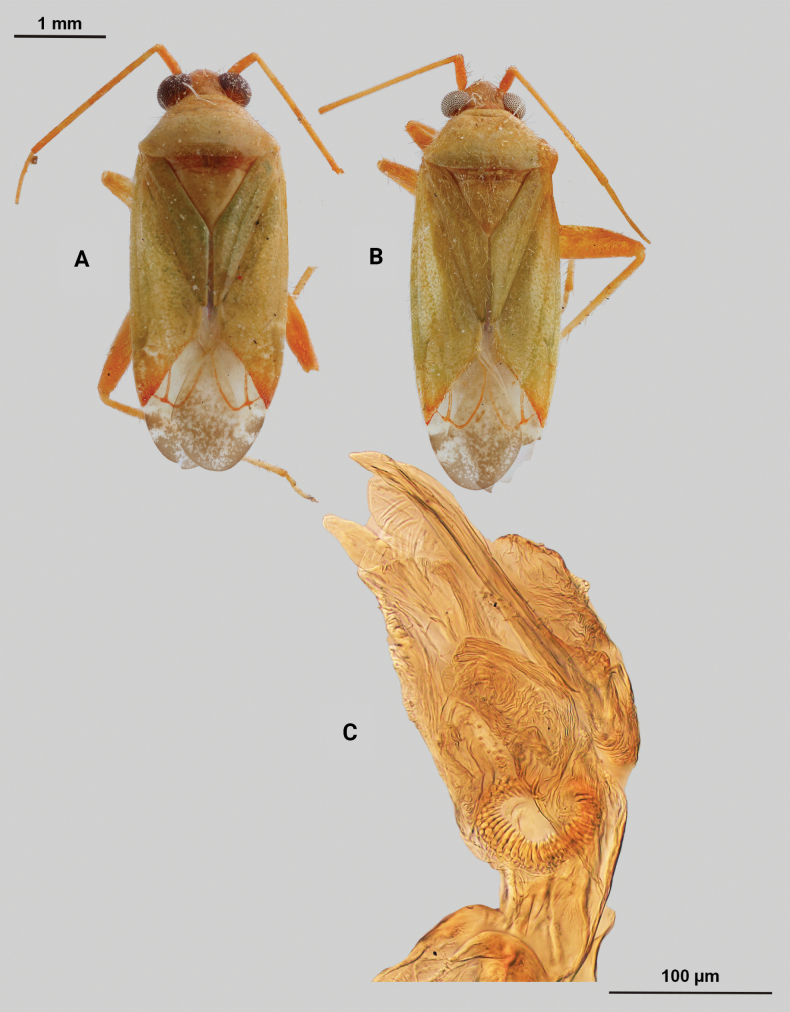
*Phytocorisvanduzeei***A** male dorsal habitus **B** female dorsal habitus **C** endosoma.

#### Note.

This species is recorded on *Larreadivaricata* Cav. (Stonedahl 1988) as a host plant, which is sparsely distributed on the lower elevation areas of the county (personal observation). We found nymphs of this bug on *L.divaricata* at the Chiricahua Desert Research Institute to confirm its presence in Jeff Davis County, but to document the morphology we utilized adult specimens from the CNC from Stonedahl’s reference collection that were collected in Arizona and New Mexico.

### ﻿ Phytocoris varius

Taxon classificationAnimaliaHemipteraMiridae

Knight, 1934 (uncertain group affiliation)

E222E5A4-6322-5CEA-BB4B-5B11EC9D6010

Fig. 22

#### Specimens examined.

Davis Mountains Resort, Marqua residence, 5800 ft, 30.62842°N, 104.08360°W, UV light, 14.x.2011, EG Riley, (TAMU), X0893108 1♂, X0894003 1♂, X0894801 1♂; 10.v.1993, malaise trap, D Marqua, (TAMU), X0968174 1♀; 21.vi.2011, UV light, Quinn & Riley, (TAMU), X0620548 1♂; Davis Mountains Hwy 118, McDonald Observatory, 30°41'12"N, 104°02'04"W, 1892 m, 14.viii.2001, JC Schaffner, (TAMU), X0963246 1♀, X0962350 1♂, X096038 1♂, X0966510 1♂, X0961621 1♀, X0964260 1♂, X0966474 1♂, X0966536 1♀; Davis Mountains Resort, Marqua residence, 5800 ft, 30.62842°N, 104.08360°W, 4–5.vii.2009, E & ML Riley, UV light (TAMU), X0614513 1♂.

**Figure 22.**
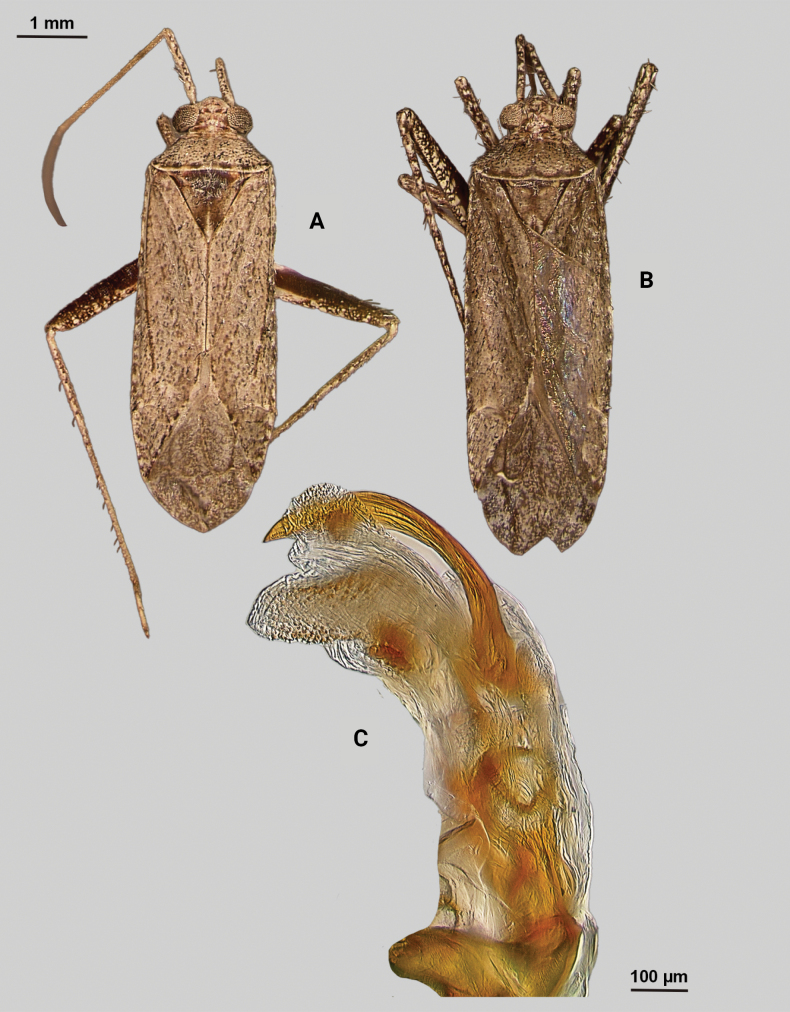
*Phytocorisvarius***A** male dorsal habitus **B** female dorsal habitus **C** endosoma.

### ﻿Key to species of *Phytocoris* Fallén currently known in Jeff Davis County, Texas

This key addresses species included in this work; there likely are additional species residing in the county that are not yet documented. Other keys to species of *Phytocoris* include Knight (1968) and Stonedahl (1988), which can be used to identify taxa that may not be included herein. While an effort was made to make this key workable using only external characters, male genitalic characters are still important for some couplets to delineate species (e.g., couplet 6), so males should be referenced if possible. For descriptions of morphological characters particularly parameres and endosomal sclerites used in this key, please refer to Stonedahl (1988).

**Table:** 

1	Head, pronotum and hemelytra completely to mostly grayish brown, brown or dark brown; no reddish tinge on any part of the wing	**2**
–	Head, pronotum, and hemelytra may possess dark brown markings but not completely dark brown; instead, mostly light brown to beige, green, white, pink, or yellow; wings can have reddish coloration or marks	**9**
2	Head and pronotum with noticeable pale line medially, extending approximately from apex of the tylus to the posterior margin of the pronotum (Fig. 8A, B; Stonedahl 1988: fig. 78a, b); pygophore with obvious tubercle dorsal to left paramere, left paramere with produced, serrate sensory lobe and flattened shaft and apex, right paramere elongate expanded and serrate dorsally and endosoma with 2 sclerotized processes (Fig. 8C; Stonedahl 1988: fig. 85a–f)	***Phytocoriscinereus***
–	Head and pronotum may be variously marked, but without obvious medial pale line as described above	**3**
3	Posterior of pronotum with pre-marginal dark brown and whitish line complete, extending along entire margin (Figs 1A, 4A, 5A, B, 11A, B, 13)	**4**
–	Posterior margin of pronotum without complete pre-marginal dark brown and whitish line or dark brown line medially thickened in form of inverted U-shape medially, not reaching lateral margins of pronotum (Figs 2A, B, 9A, B)	**8**
4	Hind femora dark brown with small white maculae widely distributed on ventral surface; no spots forming larger bands (Fig. 5A, B); pygophore with narrow tubercle dorsal to left paramere, endosoma with 2 sclerotized processes attached to membrane, right process sclerotized, left process with pair membranous sacs on distolateral margin (Fig. 5C; Stonedahl 1988: figs 212a–e, 236c)	***Phytocorisbaboquivari***
–	Hind femora dark brown with pale spots broadly forming bands that can extend onto dorsal surface (Figs 1A, 4A, B, 11A, B, 13A, B)	**5**
5	Endosoma with 1 sclerotized process (Figs 11C, 13C)	**6**
–	Endosoma with 2 sclerotized processes (Figs 1B, 4D)	**7**
6	Single right sclerotized process of endosoma narrow and elongate (Fig. 13C; Stonedahl 1988: figs 224, 236o), habitus of male and females as in Fig. 13A, B	***Phytocorismaricopae***
–	Single right sclerotized process of endosoma medially broad and roughly triangular (Figs 11C; Stonedahl 1988: fig. 135a–e), habitus of male and females as in Fig. 11A, B	***Phytocorisjuniperanus***
7	Base of cuneus with white area adjacent to fracture terminated by horizontal dark brown band (Fig. 1A); right sclerotized process of endosoma with broad base and straight, narrow apex (Fig. 1B)	***Phytocorismarqua* sp. nov.**
–	Base of cuneus with pale area adjacent to cuneal facture extending anteriorly into corium, without horizontal dark band but adjacent vertical dark bands (Fig. 4A, B); endosoma with right sclerotized process hook-like apically (Fig. 4D)	***Phytocorisschmitzi* sp. nov.**
8	Sclerotized process of endosoma comprised of 9 continuous, medially curved “teeth” (Fig. 2D)	***Phytocorismcivor* sp. nov.**
–	Sclerotized process of endosoma comprised of 4 or 5 curved apical “teeth” and ≤3 basal “teeth”, medial region of sclerite lacking “teeth” (Fig. 9C; Stonedahl 1988: fig. 105a–e)	***Phytocorisdecurvatus***
9	Hemelytron yellow, yellowish green to green	**10**
–	Hemelytron variously colored but not yellow or green	**13**
10	Apex of scutellum and sometimes posterior margin of pronotum with 2 dark spots surrounded by white rings; hemelytron mottled light green and white (Fig. 10A, B), endosoma as in Fig. 13C	***Phytocorisinterspersus***
–	Scutellum without 2 dark spots	**11**
11	Length of antennal segment I twice width of head across eyes (Fig. 19A, B), endosoma as in Fig. 19C	***Phytocorisseminotatus***
–	Length of antennal segment I less than width of head across eyes	**12**
12	Brownish yellow general coloration (Fig. 15A, B), endosoma as in Fig. 15C	***Phytocorisramosus***
–	Yellowish green general coloration (Fig. 21A, B), endosoma as in Fig. 21C	***Phytocorisvanduzeei***
13	Large, > 5.2 mm in length	**14**
–	Smaller, < 5.2 mm in length	**15**
14	Hemelytron with pink areas along corium, especially visible in males (Fig. 17A, B), endosoma as in Fig. 17C, D	***Phytocorisroseipennis***
–	Hemelytron without pinkish areas, beige to yellowish colored areas on clavus (Fig. 3A, B)	***Phytocorisrileyi* sp. nov.**
15	Hemelytron and cuneus unicolorous pale brown to pale grey with at most darkening around claval and cuneal margins	**16**
–	Hemelytron and cuneus with distinct markings and patterning (Figs 6, 7, 12, 14, 16, 20)	**17**
16	Antennal segment I slightly longer than width head (Fig. 22A, B); sclerite of endosoma relatively elongate and somewhat sinuate (Fig. 22C; Stonedahl 1988: fig. 274e)	***Phytocorisvarius***
–	Antennal segment I nearly twice as long as width head (Fig. 18A, B); sclerite of endosoma relatively squat with bulbous base and tapered apex (Fig. 18C, D; Stonedahl 1988: fig. 75d)	***Phytocorisschuhi***
17	Body with an overall reddish coloration on the head, pronotum, scutellum, legs, and parts of the hemelytron (Figs 6A, B, 12A, B)	**18**
–	Body without reddish coloration, at most hemelytron or cuneus with pink areas (Figs 14A, B, 16A, B)	**19**
18	Clavus and corium with simple setae mostly uniform in length, not coarse or bristle-like; hind femora mostly dark red with minute maculae; veins on wing membrane dark red; posterior pronotum with white margin (Fig. 6A, B), endosoma as in Fig. 6C	***Phytocorisberbericola***
–	Clavus and corium with long, erect bristle-like setae; hind femora with dark reddish brown and pinkish white bands in addition to minute white maculae; veins on wing membrane light brown to pink; posterior of pronotum without white margin (Fig. 12A, B), endosoma as in Fig. 12C	***Phytocorislisti***
19	Cuneus dark brown from cuneal fracture to cuneal apex; no pink or reddish patches or tinge on cuneus (Fig. 16A, B), endosoma as in Fig. 16C, D	***Phytocorisrelativus***
–	Cuneus with pinkish tinge or pink patches (Figs 7A, 20A)	**20**
20	Scutellum unicolorous yellowish light brown (Fig. 14A, B); endosoma with serrate sclerotized process (Fig. 14C); right paramere distinctively elongate (Fig. 14D)	***Phytocorisolseni***
–	Scutellum with at least 2 colors; endosoma without serrate sclerotized processes; right parameres not distinctly elongate	**21**
21	Dorsal surface of hemelytra with white and black scale-like setae intermixed with dark brown simple setae; posterior margin of pronotum with dark brown and white band (Figs 20A, B); endosoma with 1 sclerotized process (Fig. 20C)	***Phytocorissimulatus***
–	Dorsal surface without scale-like setae, but with silvery sericeous setae and black simple setae (Fig. 7A–C); posterior margin of pronotum without differing bands of color; endosoma with 2 sclerotized processes (Fig. 7D)	***Phytocoriscarnosulus***

## Supplementary Material

XML Treatment for Phytocoris marqua

XML Treatment for Phytocoris mcivor

XML Treatment for Phytocoris rileyi

XML Treatment for Phytocoris schmitzi

XML Treatment for Phytocoris baboquivari

XML Treatment for Phytocoris berbericola

XML Treatment for Phytocoris carnosulus

XML Treatment for Phytocoris cinereus

XML Treatment for Phytocoris decurvatus

XML Treatment for Phytocoris interspersus

XML Treatment for Phytocoris juniperanus

XML Treatment for Phytocoris listi

XML Treatment for Phytocoris maricopae

XML Treatment for Phytocoris olseni

XML Treatment for Phytocoris ramosus

XML Treatment for Phytocoris relativus

XML Treatment for Phytocoris roseipennis

XML Treatment for Phytocoris schuhi

XML Treatment for Phytocoris seminotatus

XML Treatment for Phytocoris simulatus

XML Treatment for Phytocoris vanduzeei

XML Treatment for Phytocoris varius

## ﻿Additional information

### Author ORCIDs

Katrina L. Menard https://orcid.org/0000-0003-0443-8440

### Data availability

All of the data that support the findings of this study are available in the main text.
